# A spontaneous multifunctional hydrogel vaccine amplifies the innate immune response to launch a powerful antitumor adaptive immune response

**DOI:** 10.7150/thno.58173

**Published:** 2021-05-08

**Authors:** Xiuqi Liang, Lu Li, Xinchao Li, Tao He, Songlin Gong, Shunyao Zhu, Miaomiao Zhang, Qinjie Wu, Changyang Gong

**Affiliations:** State Key Laboratory of Biotherapy and Cancer Center, West China Hospital, Sichuan University, Chengdu, 610041, P. R. China.

**Keywords:** spontaneous multifunctional hydrogel, Ncom Gel vaccine, cancer immunotherapy, innate immunity, adaptive immune response

## Abstract

Substantial progress has been made with cancer immunotherapeutic strategies in recent years, most of which mainly rely on enhancing the T cell response. However, sufficient tumor antigen information often cannot be presented to T cells, resulting in a failed effector T cell response. The innate immune system can effectively recognize tumor antigens and then initiate an adaptive immune response. Here, we developed a spontaneous multifunctional hydrogel (NOCC-CpG/OX-M, Ncom Gel) vaccine to amplify the innate immune response and harness innate immunity to launch and maintain a powerful adaptive immune response.

**Methods:** Ncom Gel was formed by a Schiff base reaction between CpG-modified carboxymethyl chitosan (NOCC-CpG) and partially oxidized mannan (OX-M). The effects of the Ncom Gel vaccine on DCs and macrophages *in vitro* and antigen-specific humoral immunity and cellular immunity* in vivo* were studied. Furthermore, the antitumor immune response of the Ncom Gel vaccine and its effect on the tumor microenvironment were evaluated.

**Results:** The Ncom Gel vaccine enhanced antigen presentation to T cells by facilitating DC uptake and maturation and inducing macrophages to a proinflammatory subtype, further leading to a T cell-mediated adaptive immune response. Moreover, the innate immune response could be amplified via the promotion of antigen-specific antibody production. The Ncom Gel vaccine reversed the tumor immune microenvironment to an inflamed phenotype and showed a significant antitumor response in a melanoma model.

**Conclusions:** Our research implies the potential application of injectable hydrogels as a platform for tumor immunotherapy. The strategy also opens up a new avenue for multilayered cancer immunotherapy.

## Introduction

Approaches harnessing the immune system to target tumors have attracted widespread attention in recent years 1-4. Substantial progress has been made in the clinic with immune checkpoint inhibitors, such as programmed cell death protein 1 or programmed cell death ligand 1 (PD-1/PD-L1) 5-7. However, only a minority of patients benefit from these therapies, which is likely because most therapies mainly focus on enhancing T cell function by targeting inhibitory pathways of effector T cells (Teffs) 4,8,9. However, the onset and maintenance of the T cell response are not autonomous and highly depend on innate immune cells 10. Innate immune cells, distributed extensively in the body, first detect invading antigens and present antigen information to downstream cells, thereby launching adaptive immune responses 11,12. Meanwhile, these cells can mount their own immune response, such as antibody-dependent cellular phagocytosis (ADCP) and antibody-dependent cellular cytotoxicity (ADCC) induced by antibodies and resulting in cytotoxicity or phagocytosis by macrophages or NK cells 13,14. Given these positive roles of innate immune cells in cancer immunotherapy, harnessing the innate immune response provides novel insights into malignant tumor treatments.

Innate immunity comprises various cells, such as dendritic cells (DCs), macrophages, natural killer cells and monocytes. Among them, DCs and macrophages are the key bridges between innate immunity and adaptive immunity; these cells take up and present antigens to T cells, further priming tumor-specific CD8^+^ T cells through major histocompatibility class I/II (MHC-I/II) molecules 15,16. Meanwhile, by producing cytokines, such as IL-6 and TNF-α, the innate immune response is be amplified, and the adaptive immune response is elicited [17.18]. The maturation and activation of DCs is extremely important for the occurrence of innate immune responses and adaptive immune responses 19. Moreover, redundant pathways of immune responsiveness could increase the probability of successfully dealing with antigens 20,21. Therefore, we hypothesize that a novel vaccine with multiple stimulation capabilities can amplify the innate immune response and induce a T cell-mediated adaptive immune response.

With the development of material science and biomedical technology, a variety of delivery systems have been investigated for cancer immunotherapy, including nanoparticles, liposomes, carbon nanorods and synthetic scaffolds, which provide various platforms for tumor vaccines 22-27. Hydrogels, as tumor vaccine delivery systems, have gained much attention for the following advantages: (1) As carriers, hydrogels can not only load small molecular drugs but also load a variety of immunomodulators, such as antibodies, tumor antigens, cytokines and adjuvants, which could work together to enhance the antitumor immune response [28.29]; (2) Hydrogel systems can protect active immunomodulators from degradation *in vivo*, leading to improved therapeutic bioavailability and (3) Under the encapsulation of smart hydrogel systems, the release behaviors of active substances can be controlled temporally and spatially, enhancing the immune stimulation of the vaccine 30-33.

**Scheme 1:**
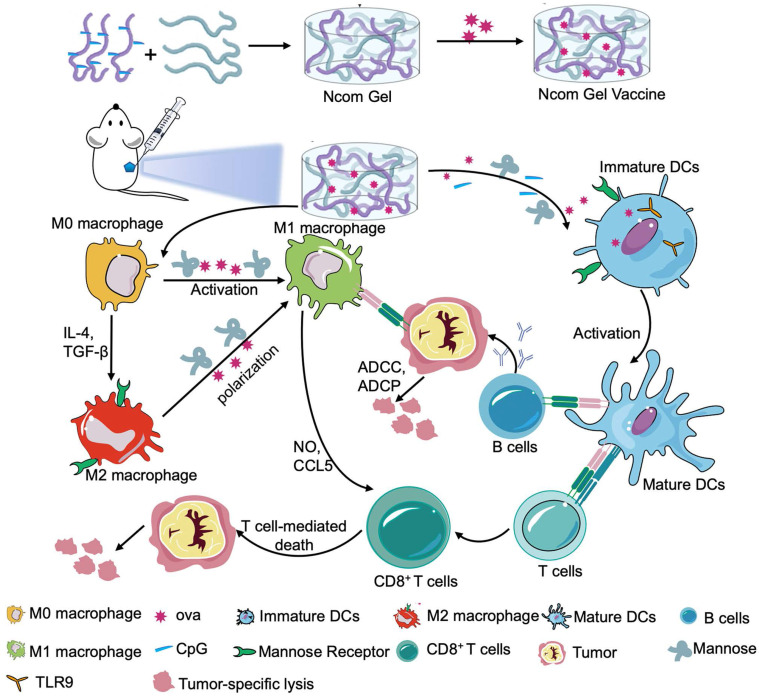
A spontaneous multifunctional NOCC-CpG/OX-M hydrogel (Ncom Gel) vaccine was designed to harness innate immunity against tumors. The innate immune response was amplified through the activation of DCs and macrophages after Ncom Gel vaccine administration. In addition, innate immune cells launched and maintained a powerful adaptive immune response through the function of the Ncom Gel vaccine.

Herein, we designed a spontaneous multifunctional NOCC-CpG/OX-M hydrogel (Ncom Gel) vaccine with multiple stimulation capabilities to amplify the innate immune response and harness innate immunity to launch and maintain a powerful adaptive immune response **(Scheme 1)**. The Ncom Gel vaccine enhances DC cell uptake and activation through multiple agonists of the mannose receptor (MR) and Toll-like receptor 9 (TLR 9) and polarizes macrophages to proinflammatory M1 subtypes. Furthermore, the Ncom Gel vaccine exhibits a distinct antigen-specific humoral immune response and antigen-specific cellular immune response, consequently showing powerful tumor inhibition in the B16F10-OVA tumor model. These results demonstrate that innate immunity is amplified after Ncom Gel vaccine administration and that the adaptive immunity response is further enhanced, inducing the dependence of T cell-mediated cytotoxicity. Our work confirms that it can be more effective to design a vaccine with multiple stimulation capabilities than a vaccine that activates a single stimulus. Additionally, our research implies the potential application of injectable hydrogels as a platform for tumor immunotherapy. The strategy also opens up new avenues for multilayered cancer immunotherapy.

## Methods

### Materials, cells and animals

Mannan from Saccharomyces cerevisiae and sodium periodate (NaIO_4_) were purchased from Sigma-Aldrich (Saint Louis MO, USA). N,O-Carboxymethyl chitosan (NOCC; degree of carboxylation 80%) was obtained from Meilunbio (Dalian, China). N-(3-dimethylaminopropyl)-N'-ethylcarbodilmide hydrochil oride (EDCI) and N-hydroxysuccinimide (NHS) were obtained from Sigma-Aldrich (Saint Louis MO, USA). 3'-NH2-C7-CpG ODN1826 (5'-TCCATGACGTTCCTGACGTT-3') was recruited from Sangon Biotech (Shanghai, China). The EndoFit ovalbumin (OVA) protein was synthesized by InvivoGen (San Diego, USA). OVA-FITC and OVA-RBITC were obtained from Beijing Biosynthesis Biotechnology Co., Ltd. (Beijing, China). Female C57BL/6 mice and BALB/c mice (6-8 weeks) were purchased from HUAFUKANG BIOSCIENCE (Beijing, China). All animal experiments were conducted according to the experimental guidelines of the Animal Experimental Ethics Committee of Sichuan University. The murine dendritic cell line DC 2.4, mouse fibroblasts L929 and Raw264.7 macrophages were provided by the American Type Culture Collection (ATCC; Manassas, VA, USA). NIH 3T3 mouse embryonic fibroblasts were obtained from the National Institutes of Health. The OVA-transfected B16F10 melanoma cell line (B16F10-OVA) was provided by the Wenzhou Institute of Biomaterials and Engineering. DC 2.4, L929 and B16F10-OVA cells were cultured in RPMI-1640 supplemented with 10% fetal bovine serum (FBS, Caoyuanlvye Bioengineering Materials Co., Ltd. Huhehaote, China). NIH 3T3 and RAW264.7 cells were cultured in DMEM supplemented with 10% FBS. The list of antibodies used for flow cytometric analysis is shown in **Table S1.**

### Preparation and characterization of oxidized mannan (OX-M)

The vicinal diol of mannan was oxidized using NaIO_4_ 34. Briefly, 300 mg (1 mmol) mannan was dissolved in phosphate buffer (pH 6.8). Sodium periodate (NaIO_4_) solution was added dropwise in the dark. Then, the solution was stirred for 2 h at room temperature. After 2 h, ethylene glycol was added to quench the reaction. The product was purified by exhaustive dialysis (MWCO 3,500) against distilled water for 3 days. The degree of oxidation was determined by hydroxylamine hydrochloride potentiometric titration. Then, 100 mg of oxidized mannan (OX-M) was added to 25 mL of hydroxylamine hydrochloride-methyl solution (0.25 mol/L). After sufficient stirring, the mixture was saturated with 0.1 mol/L NaOH solution. The degree of oxidation was calculated using the following equation: w = C_(NaOH)_ × V_(NaOH)_/6.48×10^-4^/2 ×100%.

### Synthesis and Characterization of NOCC-CpG

EDCI and NHS were used as catalyst systems to activate the carboxyl group, and 3'-NH2-C7-CpG was reacted with the intermediate product by amidation. The grafting ratio of CpG on NOCC can be controlled by the activation degree of the carboxyl group. Briefly, 22 mg NOCC was dissolved in 2-(N-morpholino) ethanesulfonic acid (MES) buffer (0.1 M, pH=6.5), EDCI (21.85 μg) and NHS (13.12 μg) were added to the mixture by diluting the stock solution. After stirring the solution for 2 h at room temperature, 10 OD 3'-NH2-C7-CpG (330 mg) was added to the reaction system and reacted for 48 h at room temperature. Exhaustive dialysis (MWCO 12,000-14,000) was performed for three days for purification. The content of CpG conjugated to the NOCC polymer was quantified with a NanoDrop 2000 (Thermo Scientific). The theoretical grafting degree of CpG on NOCC was 8 mg per milligram, and the actual grafting degree is shown in**Table S2**.

### Preparation and characterization of the Ncom Gel and Ncom Gel vaccines

NOCC-CpG (20 mg/mL) and OX-M (20 mg/mL) were dissolved in phosphate buffer. After being dissolved, the NOCC-CpG and OX-M solutions were mixed to form the Ncom Gel. For the Ncom Gel vaccine, OVA (20 μg) was first added to OX-M solution (50 μL) and further reacted with NOCC-CpG solution (50 μL). The Ncom gel and OVA/Ncom gel strengths were measured by a rheometer (Thermo Scientific). The morphology of the Ncom Gel was characterized by scanning electron microscopy (SEM). Briefly, NOCC-CpG and OX-M were crosslinked at 37 °C. Then, the formed hydrogel was freeze-dried in a lyophilizer. The dried product was cryo-fractured in liquid nitrogen. Before observation with a scanning electron microscope (JSM-5900LV, JEOL, Japan), the cross-sectional surface was coated with a thin layer of gold, and the surface and cross-sectional morphology was viewed.

### Cytotoxicity assessment of Ncom Gel

L929 and NIH 3T3 cells were seeded into 96-well plates and then incubated with different concentrations of NOCC-CpG or OX-M and various concentration solutions after immersion in 1640 or DMEM. The cells were incubated for 24 h and then incubated with MTT solution (5 mg/mL, 20 μL/well) for another 4 h. Finally, the medium was removed and replaced with 150 μL DMSO to dissolve the bottom crystal. The MTT assay was performed at 560 nm under a microplate reader. To explore the degradation behavior of Ncom Gel, Balb/c mice were subcutaneously injected with 100 μL Ncom Gel in the right back and euthanized on the 1^st^ day, 10^th^ day and 20^th^ day.

### The release behaviors of OVA-FITC from Ncom Gel

To determine the OVA release behaviors from the Ncom Gel, OVA-FITC was used and FITC fluorescence was used to measure release. Briefly, 1 mL Ncom Gel containing 200 μg OVA-FITC was prepared and sustained in PBS at 37 °C. At 0.5 h, 1 h, 2 h, 4 h, 8 h, 16 h, 24 h and 48 h, the PBS solutions were exchanged for fresh PBS medium. Meanwhile, a standard curve between the OVA-FITC concentration and the emission intensity was established. Finally, the content of OVA-FITC was measured by Gen5 (Bio-Tek, excitation wavelength, 488 nm, emission wavelength, 520 nm).

### Cellular uptake in DC 2.4 cells

DC 2.4 cells were resuspended at a density of 1 ×10^6^ cells/mL in 1640 medium and then seeded into 24-well plates. Rhodamine-labeled OVA (OVA-RBITC, 2.5 µg/mL) and 100 µL hydrogel loaded with OVA-RBITC (OVA-RBITC/Ncom Gel) were coincubated with DC 2.4 cells in the dark at 37 °C for 6 h and 8 h, respectively. Subsequently, the percentage of OVA-RBITC-positive DC 2.4 cells was determined by flow cytometry (FACS Calibur, BD, USA).

DC 2.4 cells were cultured on diameter glass coverslips in 6-well plates at a density of 5 × 10^5^ cells/well, incubated at 37 °C for 12 h and cocultured with OVA-RBITC and OVA-RBITC/Ncom Gel for 6 h. Next, the cells were labeled with LysoTracker® Green (Invitrogen, San Diego, USA) and DAPI (Beyotime, Beijing, China). Finally, the cells were washed twice and observed by a Leica multiphoton confocal microscope.

### Evaluation of bone marrow-derived dendritic cell (BMDC) maturation by flow cytometry *in vitro*

BMDCs were generated from the rear limbs of 6-to 8-week-old mice (C57BL/6). The bone marrow cells were flushed out with serum-free RPMI-1640 medium and filtered through a cell strainer to obtain a single cell suspension. Next, red blood cell lysis buffer (Hybri-Max, Sigma-Aldrich, Saint Louis MO, USA) was added to lyse the red blood cells. The remaining cells were washed twice with RPMI-1640 medium and then cultured in 1640 medium supplemented with 10% inactivated FBS, rMuIL-4 (10 ng/mL Prime Gene, USA), rmGM-CSF (10 ng/mL, Prime Gene, USA), and 2-mercaptoethanol (50 nM, Amresco, USA). The cultured medium was changed every 2 days. On the 7^th^ day, BMDCs were collected and seeded in plates (Nest Biotech, Wuxi, China). Then, free OVA, OVA plus CpG (OVA/CpG), OVA plus NOCC-CpG (OVA/NOCC-CpG), OVA plus OX-M (OVA/OX-M) and OVA encapsulated hydrogel (OVA/Ncom Gel) were added and the cells were incubated for 24 h. The concentrations of OVA, CpG, NOCC-CpG, OX-M and Ncom Gel were 2.5 µg/mL, 2 µg/mL, 125 µg/mL, 125 µg/mL and 250 µg/mL, respectively. After 24 h incubation, the cells were collected and washed twice with PBS. Next, PE anti-mouse CD11c, FITC anti-mouse CD80, PE-Cy 7 anti-mouse CD83 and APC anti-mouse CD86 antibodies (Biolegend, San Diego, CA, USA) were used to measure the expression of maturation-related markers on BMDCs by flow cytometry (ACEA NovoCyte, San Diego, California, USA).

### Examination of macrophage changes evoked by the Ncom Gel vaccine

To test whether the Ncom Gel vaccine could promote the differentiation of M0 macrophages to M1 macrophages *in vitro*, RAW264.7 cells were seeded in 24 plates (Nest Biotech, Wuxi, China). Then, free OVA, OVA plus CpG solution (OVA/CpG), OVA plus NOCC-CpG (OVA/NOCC-CpG), OVA plus OX-M (OVA/OX-M) and OVA encapsulated hydrogel (OVA/Ncom Gel) were added and the cells were cultured for 24 h. The formulation concentrations were equal to those used for the BMDC experiments. Next, the cells were obtained and stained with FITC anti-mouse CD80, PE-Cy7 anti-mouse CD86 and APC anti-mouse CD40 (Biolegend, San Diego, CA, USA). PE anti-mouse F4/80 was used to stain every aliquot of cells as a marker of macrophages. Finally, these cells were analyzed by flow cytometry (ACEA NovoCyte, San Diego, California, USA). To evaluate whether the Ncom Gel vaccine could reduce M2 macrophages *in vitro*, RAW264.7 cells were cultured with IL-4 for 24 h to induce M0 macrophages to differentiate into M2 macrophages. Next, free OVA, OVA plus CpG (OVA/CpG) and OVA encapsulated hydrogel (OVA/Ncom Gel) were added to cells for another 24 h incubation. Finally, the obtained cells were stained with PE anti-mouse F4/80 and APC anti-mouse CD206 (Biolegend, San Diego, CA, USA) and analyzed by flow cytometry (ACEA NovoCyte, San Diego, California, USA).

### Enzyme-linked immunosorbent assay

Enzyme-linked immunosorbent assay (ELISA) was used to detect the secretion of IL-6 and TNF-α by BMDCs, the secretion of IFN-γ by spleen cells and OVA-specific antibody levels produced by the hydrogel vaccine. Specifically, the concentrations of IL-6, TNF-α and IFN-γ were determined by ELISA kits (Dakewei, Shenzhen, China) following the manufacturer's directions. The specimen concentration was calculated according to the absorbance at 450 nm. To evaluate the effect of the hydrogel vaccine on antigen-specific humoral immunity, female C57BL/6 mice were subcutaneously immunized three times at the inguinal site with OVA (20 µg per mouse) or OVA admixed with free CpG (8 µg per mouse, OVA/CpG), alum adjuvant (100 µg per mouse, OVA/alum), and OVA (0.2 mg/mL) admixed with Ncom Gel (100 µL per mouse, OVA/Ncom Gel). At the indicated time points, retro-orbital blood was collected and the serum was harvested. Finally, the OVA antigen-specific antibody titer was tested with ELISA.

### Evaluation of the effects of the Ncom Gel vaccine on antigen-specific cellular immunity

The mice were divided into five groups, the PBS group (NS), the OVA (20 µg per mouse) mixed with free CpG (20 µg OVA plus 8 µg CpG per mouse, OVA/CpG) group, the OVA mixed with alum adjuvant (20 µg OVA plus 100 µg Alum adjuvant per mouse, OVA/Alum) group, the OVA (0.2 mg/mL) admixed with the Ncom Gel (20 µg OVA plus 100 µL Ncom Gel per mouse, OVA/Ncom Gel) group and the Ncom Gel only (100 µL Ncom Gel per mouse) group. Female C57BL/6 mice were immunized on the 1^st^, 15^th^ and 22^nd^ days. Next, spleen single-cell suspensions were obtained from immunized mice seven days after the last immunization, seeded in 24-well plates, restimulated with OVA (50 µg/mL) and incubated for 72 h. After incubation, spleen cells were centrifuged and washed twice, then the cultures were collected for IFN-γ detection. The obtained cells were stained with FITC anti-mouse CD8a antibody and FITC anti-mouse CD4 antibody (Biolegend, San Diego, CA, USA), respectively. Then, the labeled cells were fixed with paraformaldehyde for 30 min and washed twice. Then, the fixed cells were permeabilized with Triton X-100 (Sigma-Aldrich, Saint Louis MO, USA) and stained with PE anti-mouse IFN-γ antibody (Biolegend, San Diego, CA, USA). Finally, the cells were resuspended and analyzed by flow cytometry (ACEA NovoCyte, San Diego, California, USA).

### Evaluation the antitumor effect of the Ncom Gel vaccine

Female C57BL/6 mice were injected with B16F10-OVA cells subcutaneously in the right flank on the 1^st^ day. After five days, B16F10-OVA-bearing mice were randomly divided into seven groups and immunized with the following formulations: PBS (100 µL per mouse), OVA mixed with free CpG (20 µg OVA plus 8 µg CpG per mouse, OVA/CpG), OVA mixed with alum adjuvant (20 µg OVA plus 100 µg alum adjuvant per mouse, OVA/alum), OVA mixed with Ncom Gel (20 µg OVA plus 100 µL Ncom Gel per mouse, OVA/Ncom Gel), OVA mixed with NOCC-CpG (20 µg OVA plus 50 µL NOCC-CpG per mouse, OVA/NOCC-CpG), OVA mixed with OX-M (20 µg OVA plus 50 µL OX-M per mouse, OVA/OX-M), and Ncom Gel only (100 µL Ncom Gel per mouse). Immunization was implemented on the 5^th^, 10^th^ and 15^th^ days. All mice were administered subcutaneously at the inguinal site. During treatment, the tumor growth and body weight of the treated mice were observed. Tumor growth was determined as follows: tumor volume (V) = 0.5 × length × width^2^.

### Ncom Gel vaccine shifted the tumor immune microenvironment and elicited a strong systemic immune response

Female C57BL/6 mice (6 to 8 weeks) were transplanted with B16F10-OVA tumor cells subcutaneously in the right flank. After five days, B16F10-OVA-bearing mice were randomly divided into five groups and immunized with the following formulations: PBS (100 µL per mouse), OVA mixed with free CpG (20 µg OVA plus 8 µg CpG per mouse, OVA/CpG), OVA admixed with Ncom Gel (20 µg OVA plus 100 µL Ncom Gel per mouse, OVA/Ncom Gel) and Ncom Gel only (100 µL Ncom Gel per mouse). Immunization was implemented on day 5, 10 and 15. All mice were injected subcutaneously at the inguinal site. On the 17^th^ day, tumor- and tumor-draining lymph node (TDLN) single-cell suspensions were obtained from the immunized mice. Tumor single-cell suspensions were divided into five groups to stain tumor-infiltrating DCs, tumor-infiltrating T cells, tumor-associated macrophages (TAMs), Tregs and tumor-infiltrating myeloid-derived suppressor cells (MDSCs). Additionally, TDLN single-cell suspensions were divided into two groups for staining DC cell subsets. The flow cytometry strategies of gating DCs and T cells are shown in **Figure S16** and **Figure S18.**

### Statistical analysis

All data were analyzed by Graphpad Prism software. The results were analyzed by one-way analysis of variance (ANOVA) to determine significant differences for multiple-group analyses. Tumor growth curves over time were analyzed by two-way ANOVA with Bonferroni correction. Survival rates were determined by the Kaplan-Meier method and the log-rank test. Significant differences are reflected in the figures as * P < 0.05, **P < 0.01, *** P< 0.001 and **** P< 0.0001.

## Results and Discussion

### Synthesis of OX-M and NOCC-CpG

The Ncom Gel scaffolds were formed mainly by the following steps **(Figure S1-S2)**: First, synthetic oligodeoxynucleotides (ODNs) containing the unmethylated cytosine-phosphate-guanine (CpG) motif 3'-NH_2_-C_7_-CpG were linked to carboxymethyl chitosan by amidation reaction (termed NOCC-CpG) **(Figure S1)**. The grafting ratio of CpG on NOCC could be controlled by the activation degree of the carboxyl group. NOCC-CpG was characterized by ultraviolet spectrophotometry since the deoxynucleotides in the CpG motif have a strong absorption at 260 nm 35. As**Figure S3** shows**,** NOCC-CpG showed an obvious maximum absorption peak at 260 nm, which was similar to that of the CpG ODNs, while there was no clear absorption of NOCC, which demonstrated that we successfully modified the CpG motif into carboxymethyl chitosan. We further used Fourier transform infrared (FT-IR) spectroscopy to characterize the product **(Figure S4)**. A similar conclusion was obtained. Subsequently, OX-M was prepared through oxidation of the vicinal diol in mannan and characterized by *^1^H*-NMR. Serial new peaks from 4.8 ppm to 4.9 ppm on *^1^H*-NMR of OX-M demonstrated aldehyde group formation (**Figure S5).** The oxidation degree of OX-M was 10% through hydroxylamine hydrochloride titration. These results demonstrated that NOCC-CpG and OX-M were successfully synthesized.

### Preparation and characterization of the Ncom Gel

**Figure 1:**
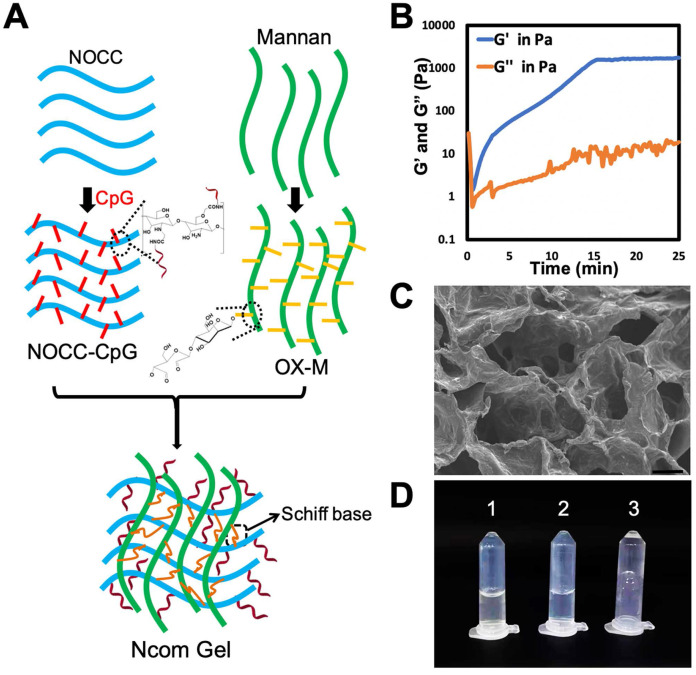
Design and characterization of the Ncom Gel. (A) Schematic showing NOCC-CpG/OX-M hydrogel formation. (B) Rheological analysis of the NOCC-CpG/OX-M hydrogel. The curves of the energy storage modulus (G') and loss modulus (G") during the crosslinking of OX-M (20 mg/mL) and CpG-b-NOCC (20 mg/mL) to hydrogels were obtained. (C) Representative SEM image of the hydrogel. Scale bar, 200 µm. (D) Morphological observation of the hydrogels (1) NOCC-CpG, (2) OX-M, and (3) Ncom Gel.

The Ncom Gel adjuvant was formed by a Schiff base reaction between the amino group of NOCC-CpG and the aldehyde motifs of OX-M **(Figure 1A)**. The intensity of the hydrogel was attributed to the count of amino groups of NOCC-CpG and aldehyde motifs of OX-M. Specifically, NOCC-CpG and OX-M were dispersed in phosphate buffer at 37°C. After being fully dissolved, the NOCC-CpG solution was mixed with OX-M solution; consequently, the reaction between amino acids and aldehydes was started, leading to the cross-linking of NOCC-CpG with OX-M. To further explore the hydrogel properties, the gelation process was observed and recorded by rheometer at 37°C. As **Figure 1B** shows, the elastic modulus (G') and viscous modulus (G") were very low at first, and the trends of G' and G" fluctuated because the system was still aqueous. Subsequently, the changes in G' were faster than those in G" over time, which demonstrated that Sciff bases between NOCC and OX-M were formed. The value of G' exceeded the G'' at approximately 36 s, which was considered the gelation of Ncom Gel. At 15 mins, G' stabilized at 1500 Pa, indicating that the hydrogel tended to be stable. We also tested the rheological behavior of the Ncom Gel by frequency sweep and found that the hydrogel had stable mechanical properties at 37°C** (Figure S6)**. Moreover, the micromorphology of the Ncom Gel was characterized by scanning electron microscopy (SEM). As **Figure 1C** shows, the hydrogel had a highly crosslinked and porous structure, which facilitated antigen adsorption. Finally, a sol-gel transition image of the Ncom Gel is presented in **Figure 1D.** NOCC-CpG and OX-M were solutions, while the Ncom Gel adhered to the EP tube wall. These data indicated that the Ncom Gel was successfully prepared. MTT assay results of the hydrogel showed that there were no obvious side effects in normal cells **(Figure S7)**. Additionally, Ncom Gel was gradually degraded* in vivo*, demonstrating its biodegradability **(Figure S8)**. To form a complete vaccine, OVA was encapsulated by Nocm Gel. The rheological curves of OVA/Ncom Gel were similar to those of Ncom Gel **(Figure S9)**. Furthermore, we explored the release behaviors of OVA-FITC by quantifying fluorescence and found that OVA was gradually released from the hydrogel **(Figure S10)**, which indicates the capacity for prolonged antigen retention *in vivo*, leading to an amplified immune effect.

### The Ncom Gel vaccine enhances antigen uptake by DC 2.4 cells

**Figure 2:**
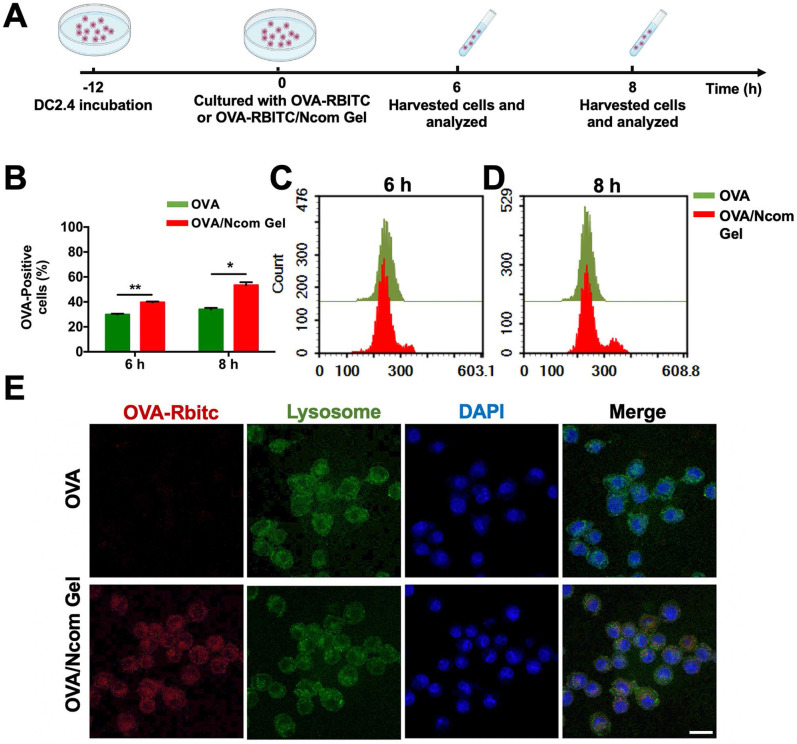
The Ncom Gel vaccine enhances the uptake of antigen by DCs. (A) Schematic illustration of DC 2.4 cellular uptake. (B) DC 2.4 cells were cultured with free OVA-RBITC or OVA-RBITC/Ncom Gel and determined by flow cytometry (n = 3, mean ± s.d., * P < 0.05, **P < 0.01, *** P< 0.001 and **** P< 0.0001). (C-D) The histograms of DCs 2.4 analyzed using flow cytometry at 6 h and 8 h. (D) DC 2.4 cells were coincubated with soluble OVA-RBITC or OVA-RBITC/Ncom Gel at 37°C for 6 h, stained with Lysol-Tracker Green and DAPI and recorded by laser scanning confocal microscopy (Scale bar= 20 µm).

Antigen presenting cells (APCs), especially dendritic cells (DCs), play a major role in the immune response. Antigens can be captured and subsequently processed and presented to T cells by DCs, inducing a downstream adaptive immune response 36. To explore the adjuvant effect of the Ncom Gel, we first investigated the effects of the Ncom Gel on antigen uptake. RBITC-labeled OVA (OVA-RBITC) was used as a model antigen. Next, DC 2.4 cells were incubated at 37 °C with free OVA and OVA/Ncom Gel groups at 6 h and 8 h, respectively** (Figure 2A)**. As** Figure 2B-C** show, the OVA-positive rates of the OVA/Ncom Gel group increased to approximately 40%, while that of the OVA group was 29%. Furthermore, as shown in **Figure 2B** and** 2D**, the OVA-positive rate of DCs 2.4 in the Ncom Gel vaccine group was up to 55.8%, which was approximately 1.6 times that in the OVA group (33%). These data demonstrated that Ncom Gel can enhance the antigen uptake of DC 2.4 cells, favoring activation of the downstream immune system. We further observed OVA-RBITC uptake by DC 2.4 cells by confocal fluorescence microscopy. In **Figure 2E**, red fluorescence signals in DC 2.4 cells treated with OVA-RBITC/Ncom Gel were higher than those with soluble OVA-RBITC, which was consistent with the flow cytometry results.

### The Ncom Gel vaccine significantly promotes the maturation of BMDCs through multiple stimuli

**Figure 3:**
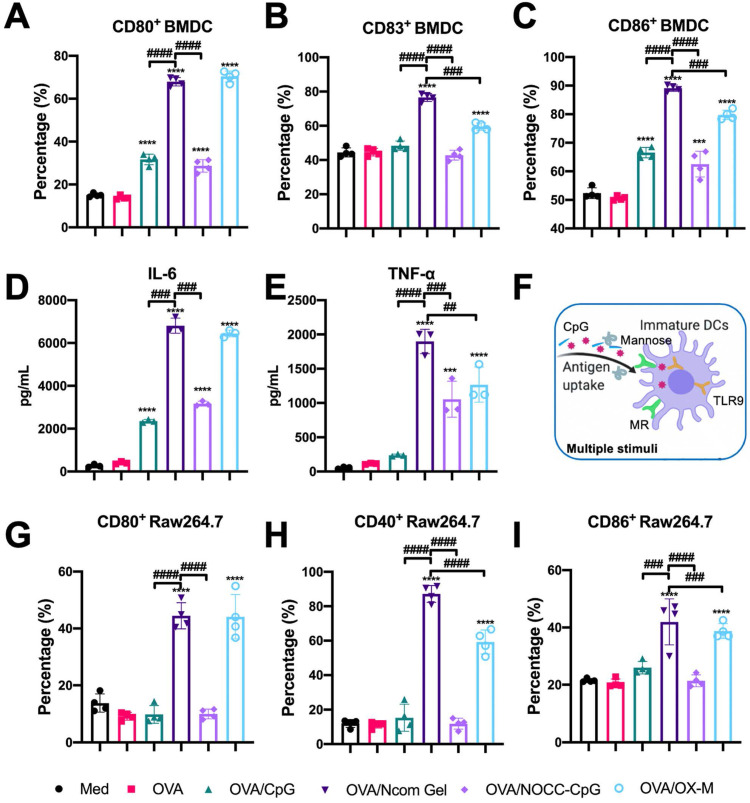
Evaluation of APC activation by the Ncom Gel vaccine *in vitro*. (A-C) Flow cytometry analysis of the expression of CD80, CD83 and CD86 on BMDCs (n=3, mean ± s.d., **P < 0.01, *** P< 0.001, *** P< 0.0001). (D-E) The secretion of IL-6 and TNF-α in BMDC suspensions detected by ELISA kits (n = 3, mean ± s.d., * P < 0.05, **P < 0.01, *** P< 0.001 and **** P< 0.0001). (F) Schematic illustration of DC activation through multiple stimuli. (G-I) The percentages of CD80^+^ Raw264.7 (A), CD40^+^ Raw264.7 (B) and CD86^+^ Raw264.7 (C) cells in various groups (n=3, mean ± s.d., * P < 0.05, **P < 0.01, *** P< 0.001 and **** P< 0.0001).

After evaluation of the effects of hydrogel vaccines on antigen uptake, we further explored the effects of hydrogel vaccines on the maturation of BMDCs. It is well known that mature BMDCs highly express costimulatory molecules such as CD80, CD83, and CD86, providing a second signal for the full activation of T cells 37. Hence, the expression of costimulatory molecules on BMDCs was examined by flow cytometry. After incubating immature BMDCs with OVA, OVA/CpG, OVA/Ncom Gel, OVA/NOCC-CpG or OVA/OX-M for 24 hours, the cultured cells were collected and analyzed **(Figure S11)**. Compared with free OVA, the OVA/Ncom Gel could significantly enhance the expression of CD80, CD86 and CD83 on DCs **(Figure 3A-C)**, suggesting that the Ncom Gel vaccine could promote immature BMDC maturation. Notably, CD80, CD86 and CD83 expression in the OVA/Ncom Gel group was higher than that in the OVA/CpG, OVA/NOCC-CpG or OVA/OX-M groups, which demonstrated that BMDCs could have greater activation through multiple pathways **(Figure 3F)**. We also investigated the secretion of cytokines during BMDC maturation. From **Figure 3D-E**, it can be observed that the Ncom Gel vaccine greatly enhanced the production of the pro-inflammatory factor IL-6 and Th1 type cytokine TNF-α, which may be attributed to the multi activation of BMDCs through MR and TLR 9. These positive phenomena suggested that the Ncom Gel vaccine could be a promising approach for amplifying the innate immune response and inducing adaptive immunity.

### The Ncom Gel vaccine induces macrophages to differentiate into proinflammatory subtypes

As another APC, the macrophage also plays an important role in recognizing tumor antigens and initiating a T cell-specific immune response. As costimulatory factors of macrophages, the upregulation of CD40, CD80 and CD86 expression indicates macrophage polarization towards the M1 proinflammatory phenotype 38-39. Therefore, we explored the effect of the Ncom Gel vaccine on costimulatory factor expression in macrophages. Free OVA, OVA/CpG, OVA/Ncom Gel, OVA/NOCC-CpG or OVA/OX-M was cocultured with RAW264.7 cells for 24 h. Next, the expression levels of CD40, CD80 and CD86 were analyzed by flow cytometry** (Figure S12)**. Compared with the other treatments, the Ncom Gel vaccine induced higher marker expression, indicating that the Ncom Gel vaccine could promote M0 macrophages to differentiate into M1 macrophages **(Figure 3G-I)**. These data demonstrated that the Ncom Gel vaccine could successfully induce macrophages to elicit an innate immune response. Furthermore, we also explored whether the Ncom Gel vaccine could reduce the level of anti-inflammatory M2 macrophages. As **Figure S13** shows, the number of M2 macrophages in the OVA/Ncom Gel-treated groups was decreased, indicating that the hydrogel vaccine can reduce the number of anti-inflammatory M2 macrophages. These data demonstrated that the Ncom Gel vaccine could positively regulate the immunity of macrophages.

### The Ncom Gel vaccine dramatically enhances the production of antigen-specific antibodies *in vivo*

**Figure 4:**
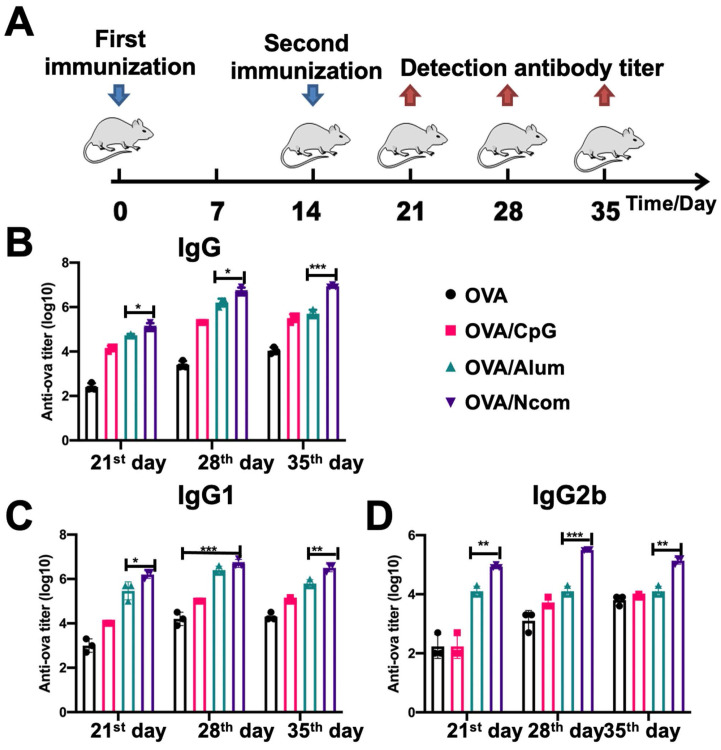
The Ncom Gel vaccine dramatically enhanced the production of antigen-specific antibodies *in vivo*. (A) Schematic illustration of immunization and analysis schedule. (B) The titer of anti-OVA IgG antibodies in plasma was detected by ELISA. (C-D) The titers of anti-OVA IgG1 antibodies and anti-OVA IgG2b antibodies in plasma on days 21, 28 and 35 were detected by ELISA (n=3, mean ± SD, * P < 0.05, **P < 0.01, *** P< 0.001 and **** P< 0.0001).

Encouraged by the results obtained with DCs and macrophages, we further investigated whether the Ncom Gel vaccine could enhance antigen-specific humoral immune responses *in vivo*, which could involve the effector response via ADCC or ADPC. We selected aluminum adjuvant as a positive control 40. Different formulations were injected subcutaneously into C57BL/6 mice. Then, OVA-specific antibody titers were detected by ELISA at the indicated time points **(Figure 4A)**. As **Figure 4B** shows, the amount of IgG produced by the OVA/Ncom Gel vaccine at different time points was distinctly higher than that produced by OVA/Alum and OVA/CpG. Specifically, on the 28^th^ day, compared with free OVA, the OVA/Ncom Gel vaccine increased IgG production by 2524-fold, while the aluminum adjuvant increased IgG production by 1049-fold. With respect to the IgG isotype, the Ncom Gel vaccine notably increased the OVA-specific levels of IgG1 and IgG2b** (Figure 4C-D)**. Meanwhile, the titers of the OVA/CpG group were higher than those of the OVA alone group, possibly due to the expression of TLR9 in B cells. These results demonstrated that the Ncom Gel vaccine could dramatically enhance antigen-specific humoral immunity *in vivo*, suggesting that Ncom Gel is an effective adjuvant for inducing innate immunity.

### The Ncom Gel vaccine significantly improves adaptive immunity *in vivo*

**Figure 5:**
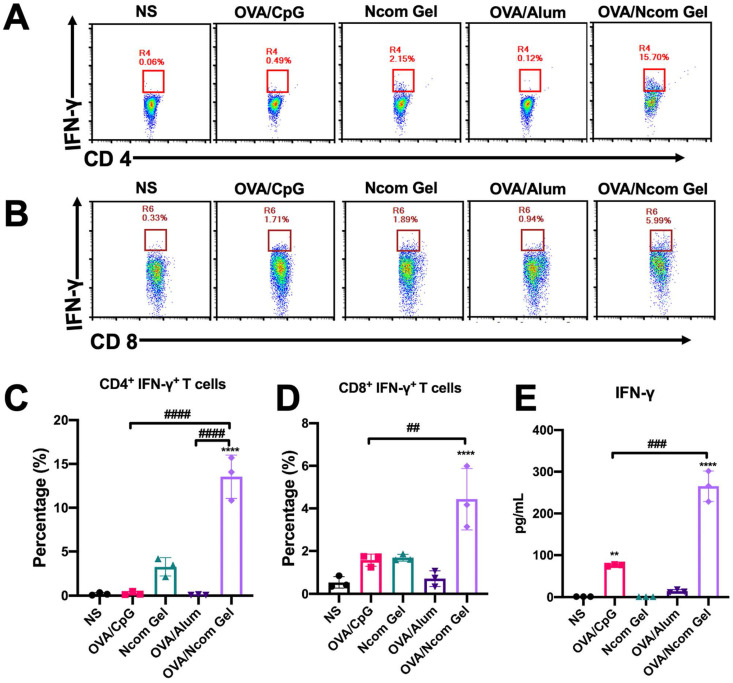
The Ncom Gel vaccine has a significant effect on the adaptive immune response *in vivo*. (A-D) Antigen-specific T cell analysis in the spleen after immunization with different formulations (A, C, CD4^+^ IFN-γ^+^ T cell analysis; B, D, CD8^+^ IFN-γ^+^ T cell analysis). (E) Detection of IFN-γ secreted by T cells by ELISA after restimulation with antigen (n=3, mean ± s.d., * P< 0.05, *** P< 0.001).

After confirming that the Ncom Gel vaccine was able to enhance the innate immune response in mice, we further explored whether the Ncom Gel vaccine could enhance adaptive immune responses. C57BL/6 mice were injected subcutaneously three times and sacrificed on the 28^th^ day. Then, the percentages of CD4^+^ IFN-γ^+^ T cells and CD8^+^ IFN-γ^+^ T cells in the spleen were analyzed by flow cytometry. As shown in** Figure 5A-B**, the levels of CD4^+^ IFN-γ^+^ T cells and CD8^+^ IFN-γ^+^ T cells were significantly increased in the OVA/Ncom Gel vaccine group. Compared with other groups, the ratios of CD4^+^ IFN-γ^+^ T cells and CD8^+^ IFN-γ^+^ T cells in the OVA/Ncom Gel vaccine group were 13.47% and 4.71%, respectively **(Figure 5C-D)**. These results validated that the Ncom Gel vaccine could effectively promote IFN-γ secretion by cytotoxic T cells *in vivo*. Furthermore, promotion of CD4^+^ IFN-γ^+^ helper T cells could facilitate the activation of CTLs and production of antiviral cytokines. We further detected related cytokines secreted by T cells after restimulation with OVA. As** Figure 5E** shows, the secretion of IFN-γ was significantly increased after Ncom Gel vaccine administration. As a Th1 cytokine, IFN-γ can not only promote the production of IgG2b but also mediate the response of CD8^+^ CTLs.

### The Ncom Gel vaccine elicits effective antitumor activity

**Figure 6:**
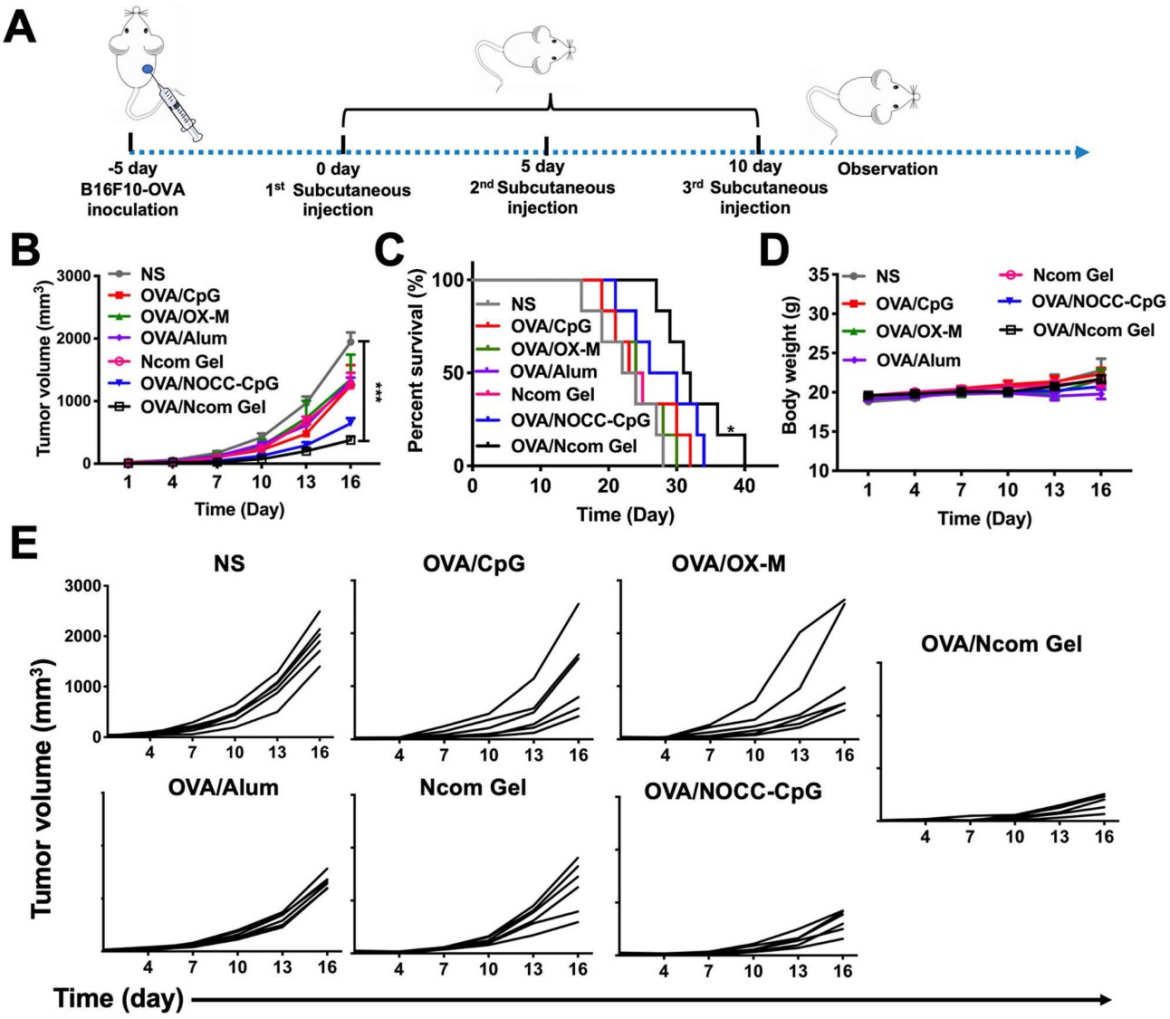
The Ncom Gel vaccine dramatically inhibits melanoma growth in C57BL/6 mice. (A) Treatment scheme for C57BL/6 mice with B16F10-OVA tumors. (B) Average tumor growth curves of B16F10-OVA tumor-bearing mice were recorded after treatment with PBS, OVA/CpG, OVA/OX-M, OVA/NOCC-CpG, OVA/Ncom Gel, OVA/alum or NcomGel (n=6; data are represented as the means ± SEM and analyzed with two-way ANOVA with Bonferroni correction tests. * P < 0.05, **P < 0.01, *** P< 0.001 and **** P< 0.0001.) (C) Survival curves of mice after the mentioned treatments. (D) Body weight curves were recorded during the treatment. (E) Individual mouse tumor growth curves in different treatment groups.

Inspired by the potent induction of the innate immune response and adaptive immune response by the Ncom Gel vaccine, we then investigated the antitumor activity induced by the vaccine. To establish a B16F10-OVA-bearing mouse model, B16F10-OVA tumor cells were injected subcutaneously into the right flank of female C57BL/6 mice. After the tumor volume reached approximately 100 mm^3^, the mice were randomly divided into seven groups and immunized with different vaccines** (Figure 6A)**. As shown in **Figure 6B** and** 6E,** the OVA/Ncom Gel group significantly inhibited tumor growth, while the free OVA, OVA/CpG, OVA/OX-M, Ncom Gel and OVA/alum groups showed no obvious inhibition. The OVA/NOCC-CpG group showed partial inhibition of melanoma, which may be due to the viscosity of the NOCC-CpG solution, resulting in prolonged antigen release in mice. Survival was substantially prolonged in C57BL/6 mice treated with OVA/Ncom Gel **(Figure 6C)**. Potential toxicity of the Ncom Gel vaccine was further detected. The body weight of the OVA/Ncom Gel group was similar to that of the other groups **(Figure 6D)**. Blood chemistry profile analysis **(Figure S14)** and H&E staining of vital organ sections** (Figure S15)** after treatment with various formulations showed no distinct difference, indicating that the Ncom Gel vaccine has no noticeable toxicity. These data implied that the hydrogel vaccine could provide a potential application to cancer immunotherapy.

### The Ncom Gel vaccine reverses the tumor immune microenvironment by eliciting a strong systemic immune response

**Figure 7:**
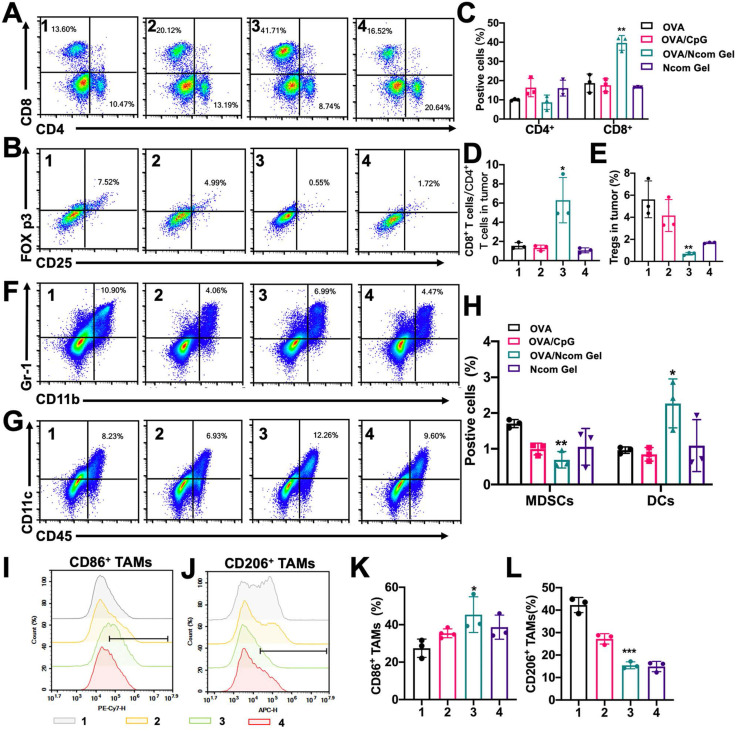
The Ncom Gel vaccine changes the tumor immune microenvironment through a strong systemic immune response. (A-B) Representative flow cytometry dot plot of tumor infiltrating CD8^+^ T cells and CD4^+^ T cells (A) and Tregs (B) 2 days following the last treatment. (C) Quantitative data of CD4^+^ T cells and CD8^+^ T cells were analyzed (n=3 biologically independent samples). (D) The ratios of CD8^+^ T cells to CD4^+^ T cells in the tumor immune microenvironment (n=3 biologically independent samples). (E) Quantitative data of Tregs were examined (n=3 biologically independent samples). (F-H) The frequencies of MDSCs and DCs in tumors (F) and representative flow cytometry dot plots were analyzed following the last treatment (n=3 biologically independent samples). (I-J) Representative flow cytometry dot histograms of M1 TAMs (CD11b^+^ F4/80^+^ CD86^+^) and M2 TAMs (CD11b^+^ F4/80^+^ CD206^+^) in tumors are shown. (K-L) The frequencies of M1 TAMs and M2 TAMs in tumors examined 2 days after the last treatment (n=3 biologically independent samples). Mice were divided into the following groups: (1) NS, (2) OVA/CpG, (3) OVA/Ncom Gel, and (4) Ncom Gel. All data are represented as means ± s.d. and analyzed with one-way ANOVA with Tukey test. * P < 0.05, **P < 0.01, *** P< 0.001 and **** P< 0.0001.

Finally, we explored the effect of the Ncom Gel vaccine on the tumor immune microenvironment in the B16F10-OVA mouse model. The percentage of infiltrating CD8^+^ T cells was significantly increased **(Figure 7A** and** 7C)**, while the percentage of Tregs was dramatically decreased **(Figure 7B** and** 7E)** after Ncom Gel vaccine treatment, demonstrating that the Ncom Gel vaccine remarkably enhanced the antitumor immune response of T cells. We further evaluated the ratio of CD8^+^ T cells to CD4^+^ T cells, which is generally considered to be an indicator of response to cancer immunotherapy and clinical outcome, and found that the Ncom Gel vaccine significantly increased this ratio **(Figure 7D)**. Additionally, the number of tumor-infiltrating DCs was remarkably augmented, while the percentage of tumor-infiltrating myeloid-derived suppressor cells (MDSCs) was decreased** (Figure 7F-H)**. More importantly, the content of M1 TAMs was increased, while that of M2 TAMs was reduced **(Figure 7I-K,** and** S17)**, which suggested that the macrophages in tumors were repolarized after Ncom Gel vaccine treatment. These results suggested that the immunocellular composition of the tumor microenvironment was reversed after treatment with the Ncom Gel vaccine, favoring the induction of a strong antitumor immune response. The subsets of DC cells in tumor-draining lymph nodes (TDLNs) were further studied. From**Figure S19**, the percentage of CD103^+^ CD8a^+^ DCs in the TDLN, as the most competent APCs for cross-priming CD8^+^ T cells, was augmented after Ncom Gel vaccine administration, suggesting that our Ncom Gel vaccine can magnify APC function. The CD86 expression of DCs in TDLNs was also increased, demonstrating that the Ncom Gel vaccine can improve DC maturation* in vivo* **(Figure S20)**. This result was consistent with the *in vitro* experiments. The percentages of CD8^+^ T cells and CD4^+^ T cells in the TDLN were also evaluated, and no obvious changes were found among the various groups** (Figure S21)**. From these data, it can be concluded that the Ncom Gel vaccine can significantly elicit a strong systemic immune response and convert the immunosuppressive tumor microenvironment into an immunostimulatory state.

## Conclusions

In summary, we developed a spontaneous multifunctional hydrogel vaccine with multiple stimulation capabilities to improve the innate immune response and adaptive immune response to cancer immunotherapy. Our results demonstrated that the Ncom Gel vaccine can enhance antigen uptake and improve the maturation of dendritic cells. Further study suggested that the Ncom Gel vaccine could induce macrophages in a positive manner. Consequently, antigen-specific antibody production and the antigen-specific adaptive immune response in mice were strongly enhanced. In B16F10-OVA tumor-bearing mice, multifunctional Ncom Gel significantly inhibited tumor growth and prolonged survival. Finally, the tumor immune microenvironment of B16F10-OVA cells was shifted by the Ncom Gel vaccine. Our research indicates that the strategy of applying redundant pathways to immune responsiveness could improve the probability of successfully dealing with tumor cells. Additionally, our research also implies the potential for the application of the injectable hydrogel as a novel adjuvant to amplify the innate immune response and consequently elicit effective adaptive immunity, which opens up a new avenue for multilayered cancer immunotherapy. We hope this work can also provide a novel platform for the development of therapeutics against other infectious diseases.

## Supplementary Material

Supplementary figures and tables.

## Abbreviations

ADCC: antibody dependent cellular cytotoxicity
ADCP: antibody dependent cellular phagocytosis
BMDCs: bone marrow derived dendritic cells
DCs: dendritic cells
ELISA: enzyme-linked immune sorbent assay
MDSCs: myeloid-derived suppressor cells
MHC-I/II: major histocompatibility class I/II
MR: mannose receptor
Ncom Gel: NOCC-CpG/OX-M hydrogel
NOCC: N, O-Carboxymethyl chitosan
OX-M: oxidized mannan
TAMs: tumor associated macrophages
TDLN: tumor-draining lymph node
Teffs: effector T cells
TLR 9: Toll-like receptor 9
