# Supplementary material for: A spontaneous multifunctional hydrogel vaccine amplifies the innate immune response to launch a powerful antitumor adaptive immune response
Source: Theranostics. 2021 May 8;11(14):6936–49. doi: 10.7150/thno.58173 (PMC8171104; doi:10.7150/thno.58173)
Supplement: Supplementary file 1 — Supplementary figures and tables. [file thnov11p6936s1.pdf]

Supplemental Files

A spontaneous multifunctional hydrogel vaccine amplifies the innate immune response to launch a powerful antitumor adaptive immune response

Xiuqi Liang+, Lu Li+, Xinchao Li+, Tao He, Songlin Gong, Shunyao Zhu, Miaomiao Zhang, Qinjie Wu*, and Changyang Gong*

X. Q. Liang, L. Li, X. C. Li, Dr. T. He, S. L. Gong, S. Y. Zhu, M. M. Zhang, Prof. Q. J. Wu, Prof, C. Y. Gong

State Key Laboratory of Biotherapy and Cancer Center

West China Hospital, Sichuan University,

Chengdu, 610041, P. R. China

+ These authors contributed equally to this work.

* To whom should be corresponded, E-mail: chygong14@163.com and cellwqj@163.com

Table S1. Antibodies list used in flow cytometric analysis

Antibody	Clone	Supplier
PE anti-mouse CD80	16-10A1	BioLegend
PE-Cy7 anti-mouse CD83	Michel-19	BioLegend
FITC anti-mouse CD86	GL-1	BioLegend
FITC anti-mouse CD80	16-10A1	BioLegend
APC anti-mouse CD86	GL-1	BioLegend
APC anti-mouse CD40	3/23	BioLegend
APC anti-mouse CD206	C068C2	BioLegend
FITC anti-mouse CD8a	53-6.7	BioLegend
PE anti-mouse IFN-γ	XMG1.2	BioLegend
PE-Cy7 anti-mouse CD3	17A2	BioLegend
FITC anti-mouse CD4	GK1.5	BioLegend
APC anti-mouse CD8a	53-6.7	BioLegend
PE anti-mouse CD11c	N418	BioLegend
FITC anti-mouse CD45	30-F11	BioLegend
FITC anti-mouse CD11b	M1/70	BioLegend
PE anti-mouse F4/80	BM8	BioLegend
PE-Cy7 anti-mouse CD86	GL-1	BioLegend
PE anti-mouse CD4	GK1.5	BioLegend
FITC anti-mouse CD25	PC61.5	BioLegend

PE-Cy5 anti-mouse Foxp3	FJK-16s	eBioscience
APC anti-mouse Gr-1	RB6-8C5	BioLegend
APC anti-mouse CD103	2E7	BioLegend
APC anti-mouse CD8a	53-6.7	BioLegend

Table S2. The grafting ratio of CpG on NOCC

Sample ID	NOCC concentration (μg/mL)	Detecting concentration of CpG (μg/mL)	Theoretical concentration of CpG (μg /mL)
S1	6000	47.179	48
S2	6000	47.952	48
S3	6000	49.014	48

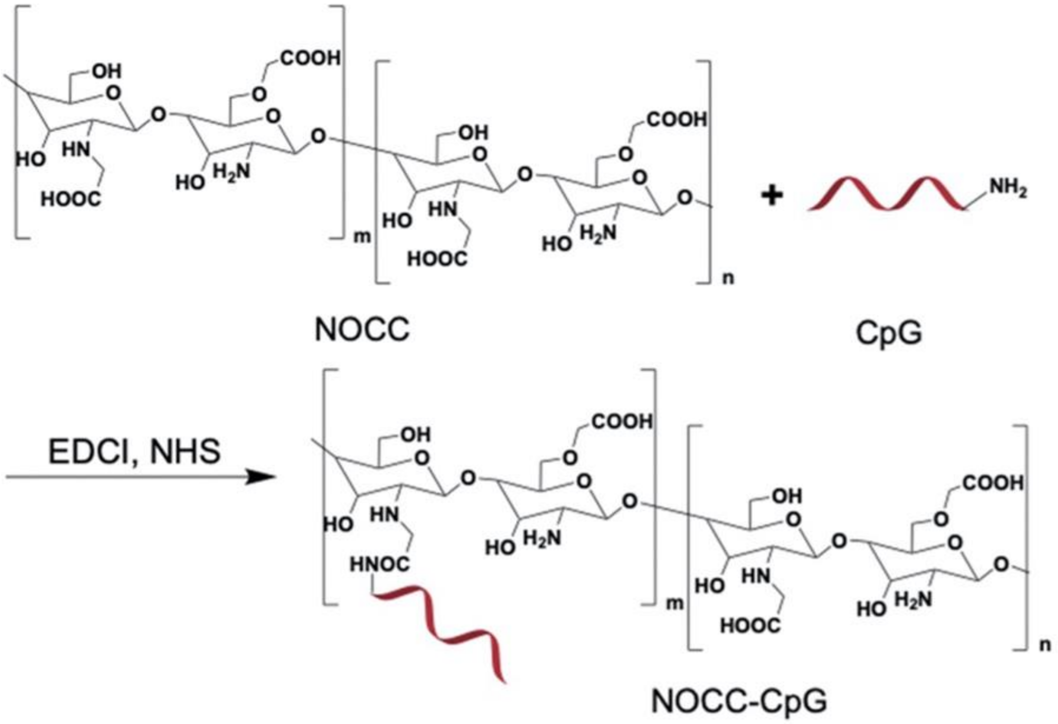

Figure S1. The scheme of synthesizing NOCC-CpG.

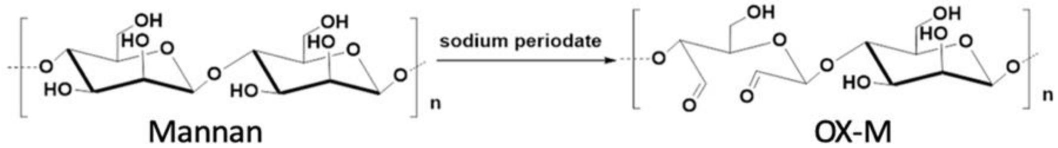

Figure S2. The scheme of synthesizing OX-M.

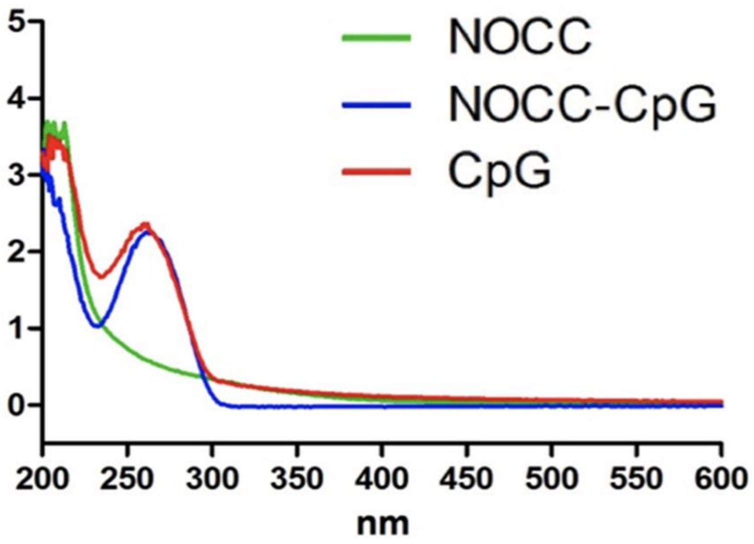

Figure S3. UV-Vis absorption spectra of CpG, NOCC, and NOCC-CpG.

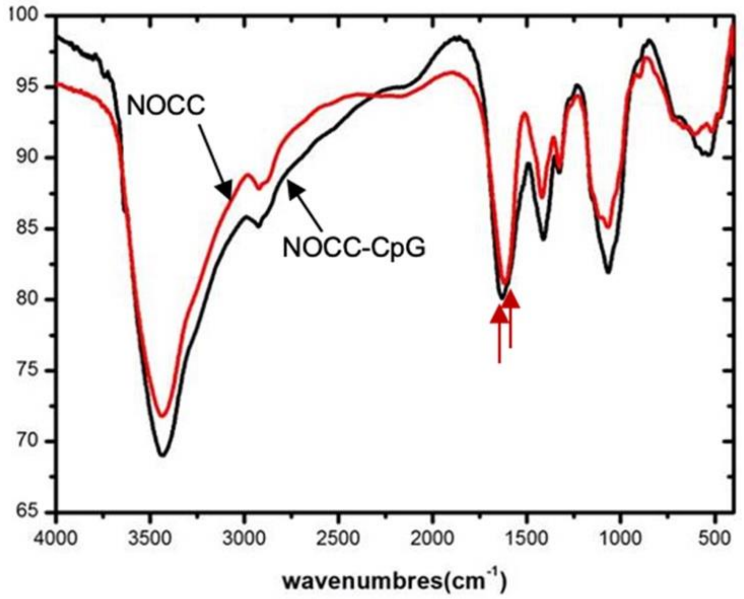

Figure S4. FT-IR spectra of the NOCC and NOCC-CpG.

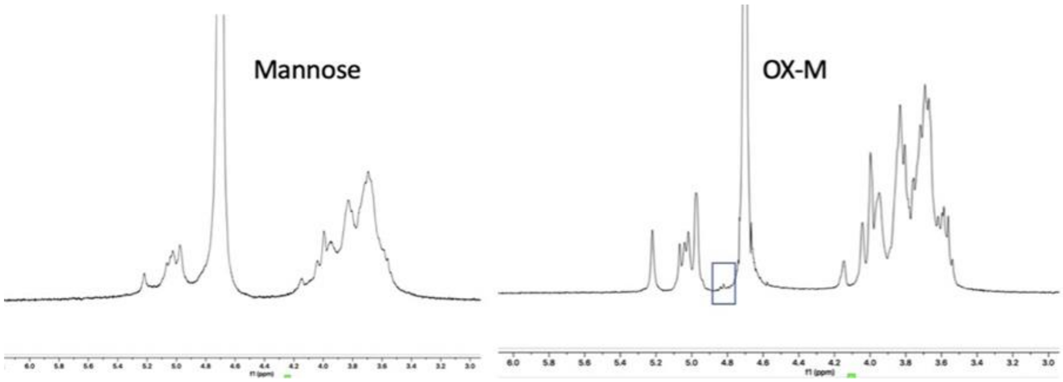

Figure S5. The ^{1}H-NMR spectra of the mannose and OX-M.

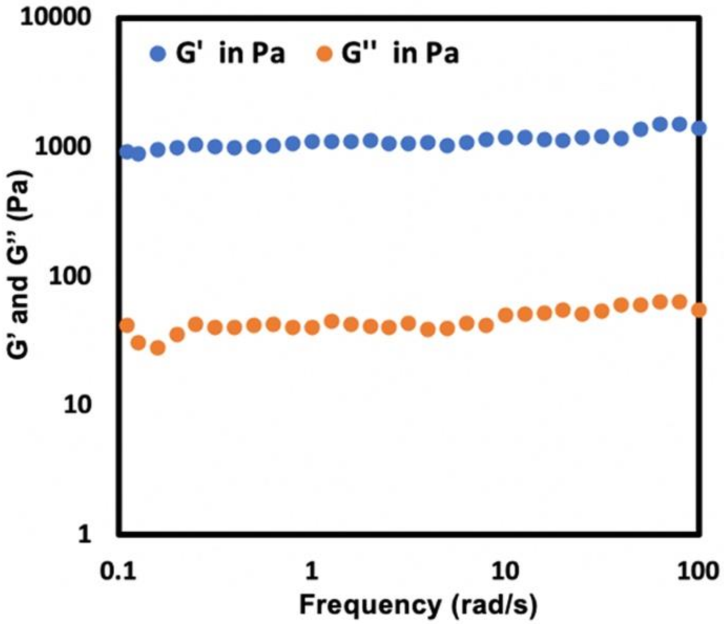

Figure S6. Dynamic frequency sweep of Ncom Gel at 37 °C.

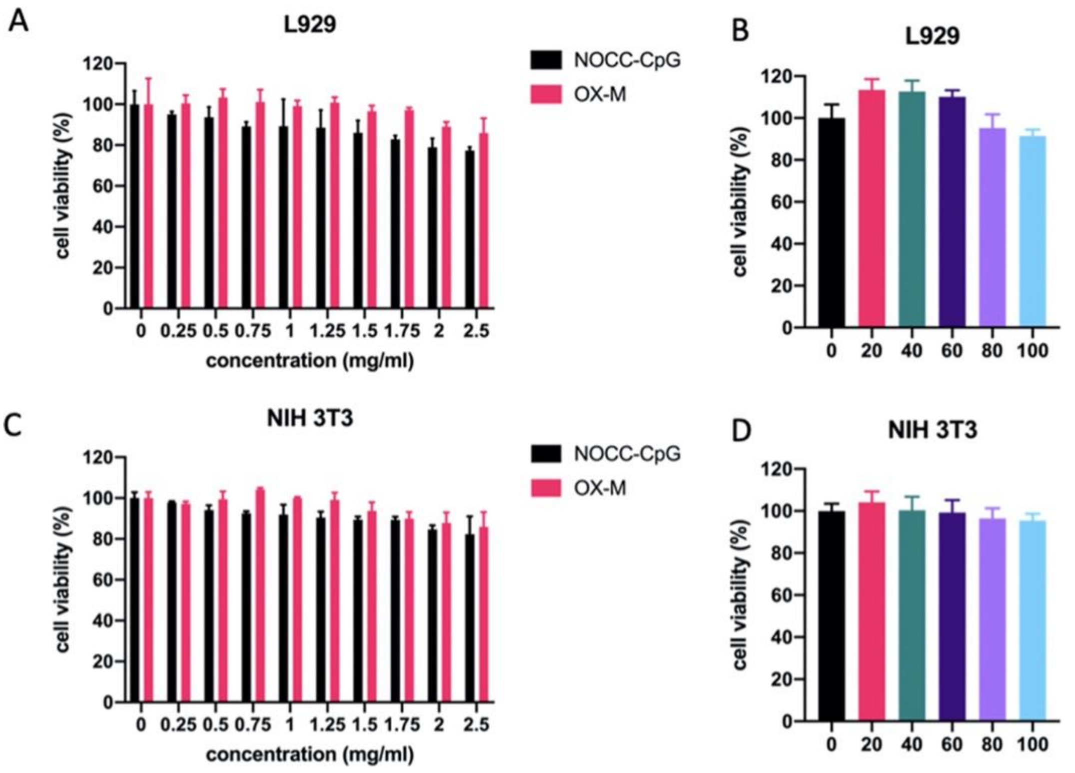

Figure S7. Cytotoxicity assessment by MTT assay for L929 and NIH3T3 cells incubated with various concentrations of NOCC-CpG and OX-M (A) and (C) and different Ncom Gel extract solutions (B) and (D) (n=3, all data are represented as means ± s.d.).

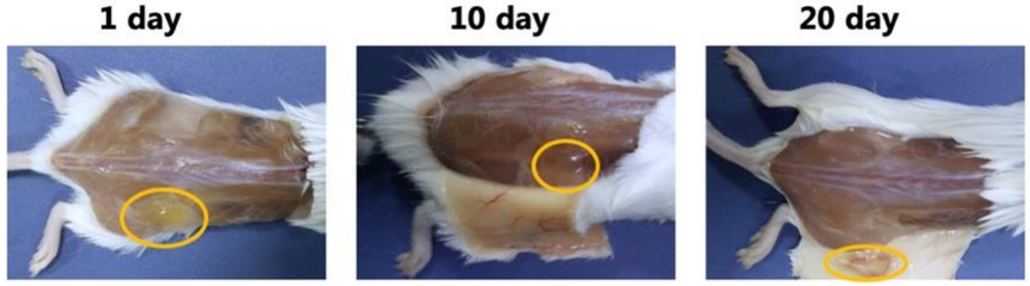

Figure S8. Evaluation of degradation behavior of Ncom Gel in mice.

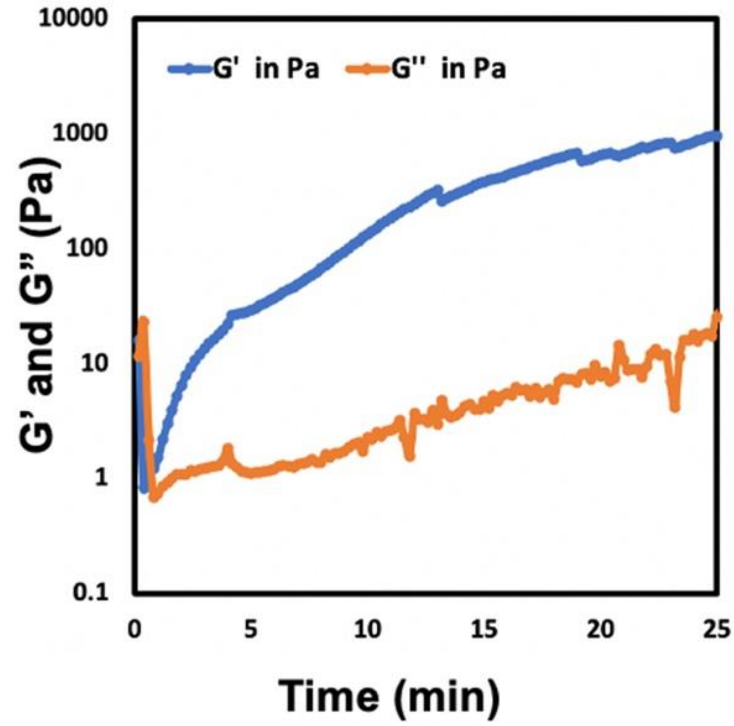

Figure S9. Rheological analysis of the NOCC-CpG/OX-M hydrogel after loading OVA (OVA/Ncom Gel).

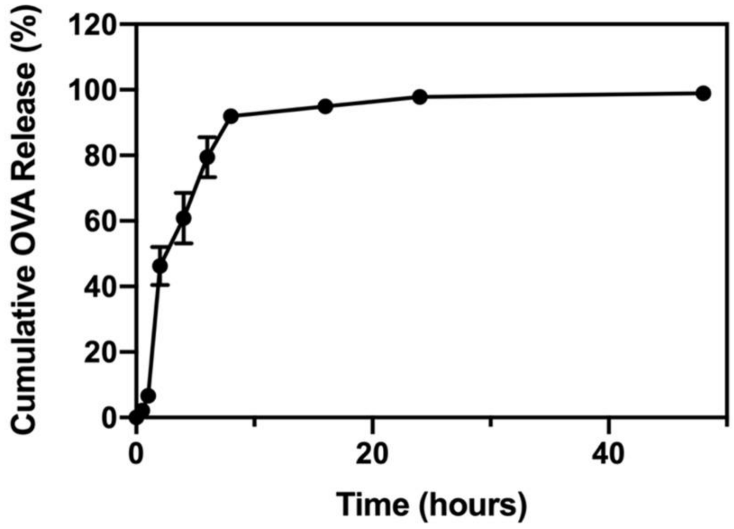

Figure S10. Cumulative release profiles of OVA-FITC from Ncom Gel in PBS.

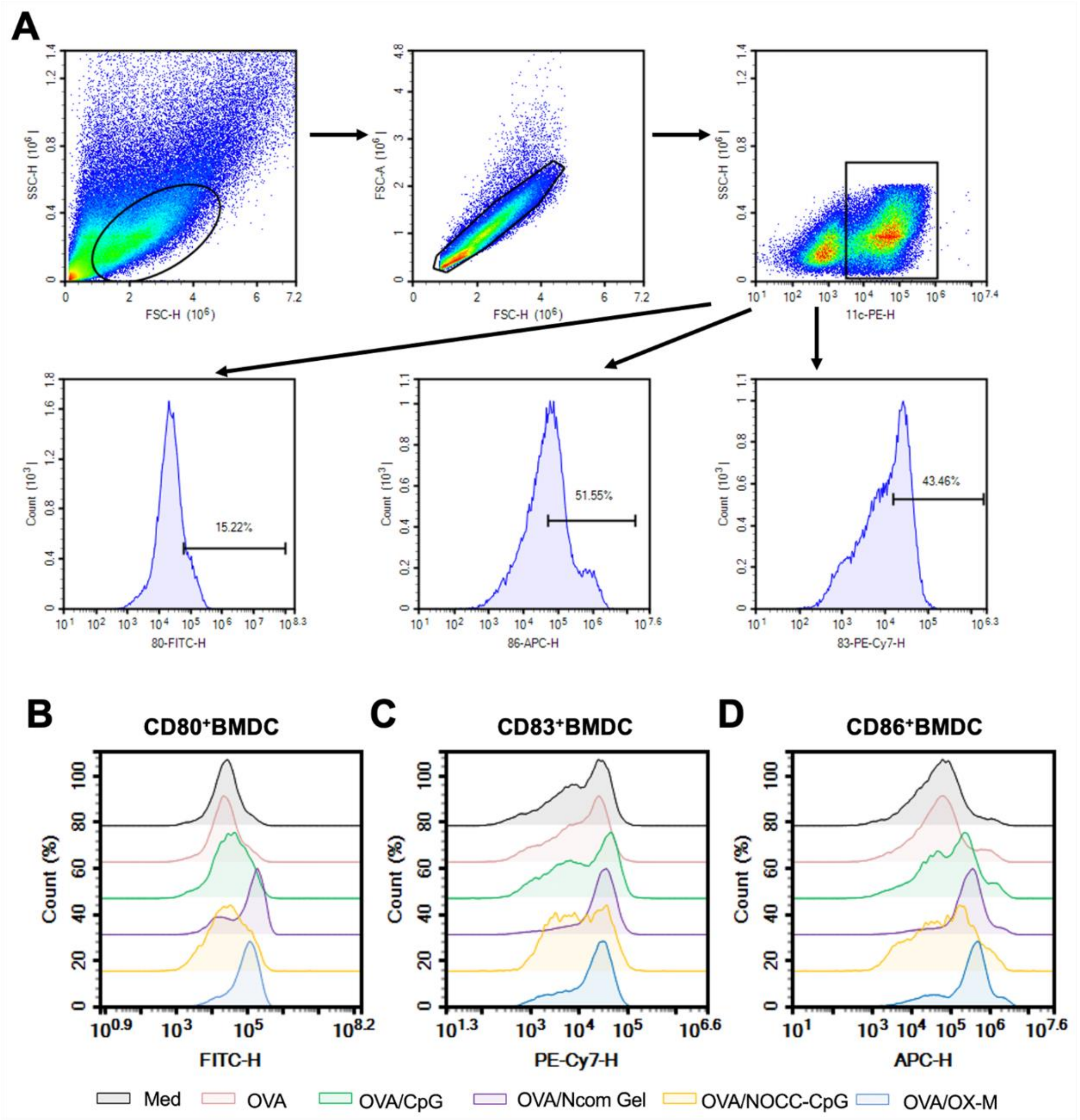

Figure S11. Evaluation of Ncom Gel vaccine on BMDC activation *in vitro.* (A) . Representative scatter plots and gating information derived from analysis of CD11c$^+$ CD80$^+$, CD11c$^+$ CD86$^+$ and CD11c$^+$ CD83$^+$ BMDCs. (B-D) Flow cytometry analysis for the expression of CD80, CD83 and CD86 on BMDCs after various treatments.

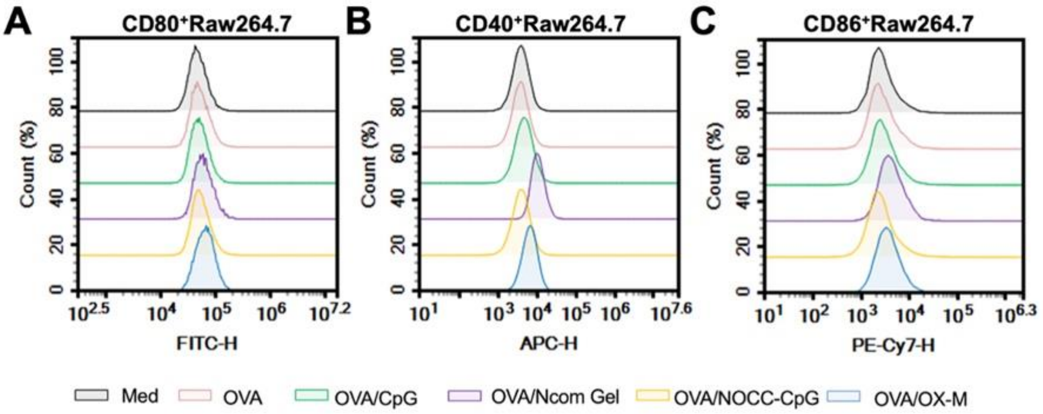

Figure S12. Evaluation of Ncom Gel vaccine on M1 polarization *in vitro.* (A-C) Flow cytometry analysis for the expression of CD80, CD40, and CD86 on Raw264.7s after different treatments.

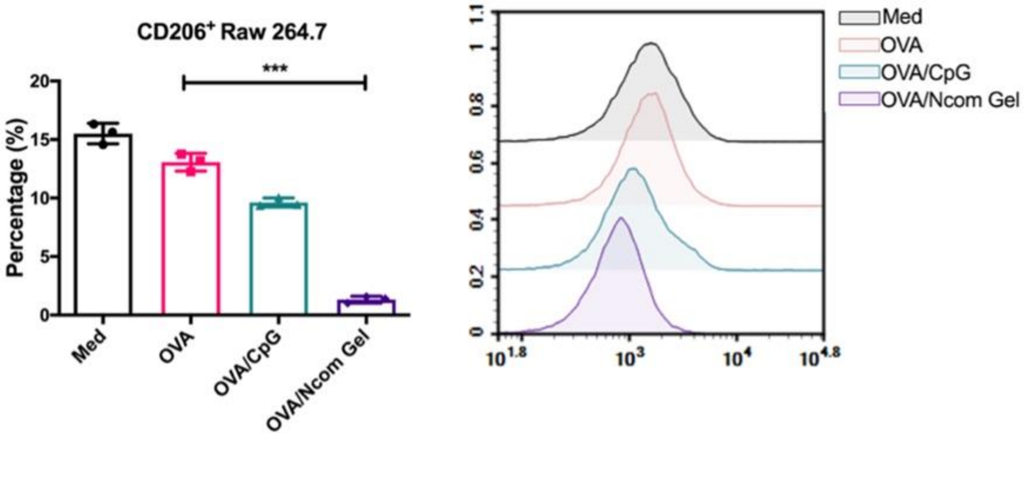

Figure S13. Evaluation of Ncom Gel vaccine on M2 polarization *in vitro.*

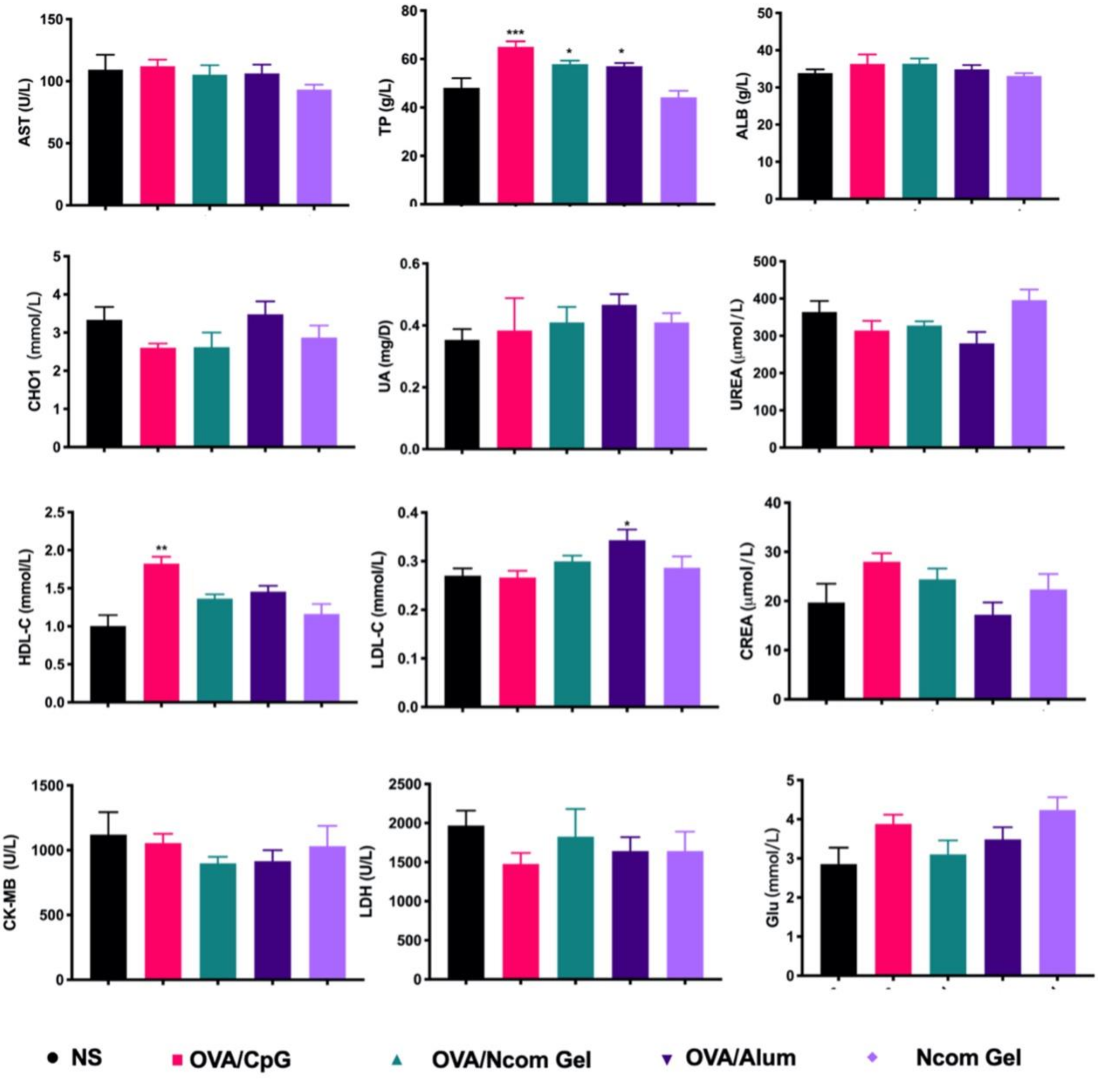

Figure S14. Blood chemistry profile analysis after treatment with various formulations (n=6 biologically independent samples, all data are represented as means ± s.d.)

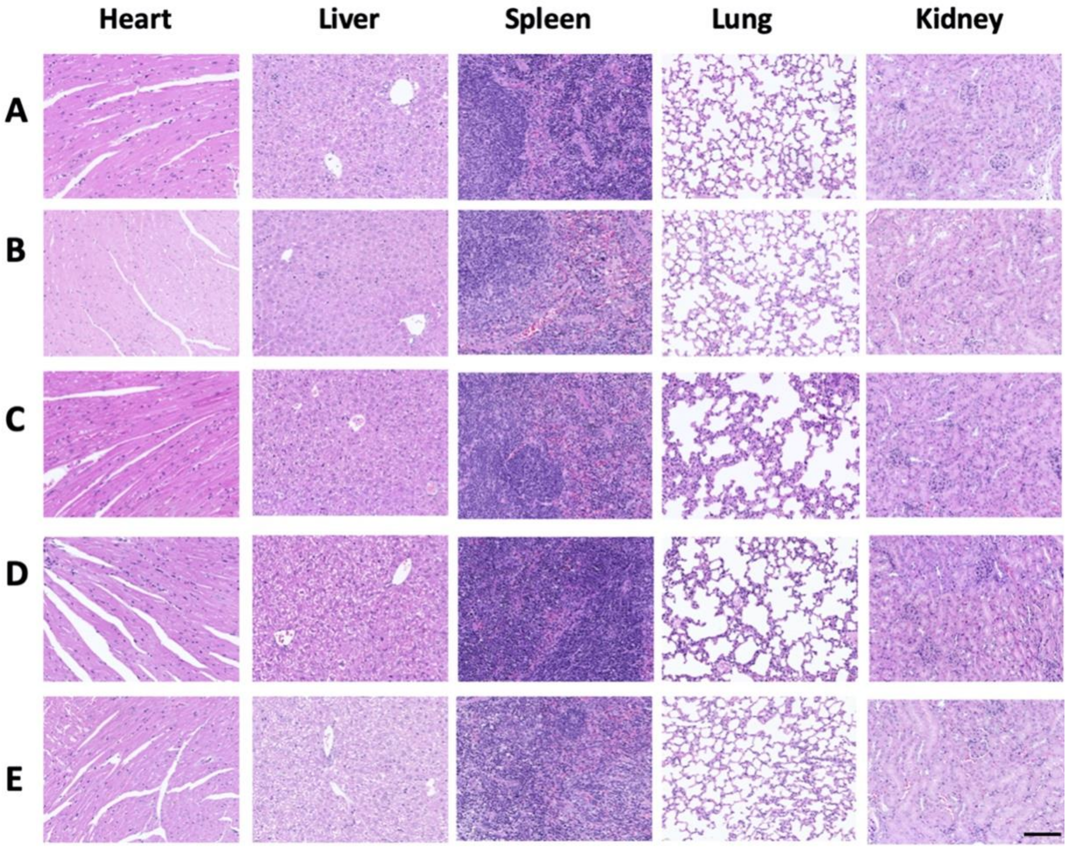

Figure S15. H&E staining of vital organ sections. PBS (A), OVA/CpG (B), OVA/Ncom Gel (C), OVA/Alum (D), and Ncom Gel (E) (Scale bar = 100 μm).

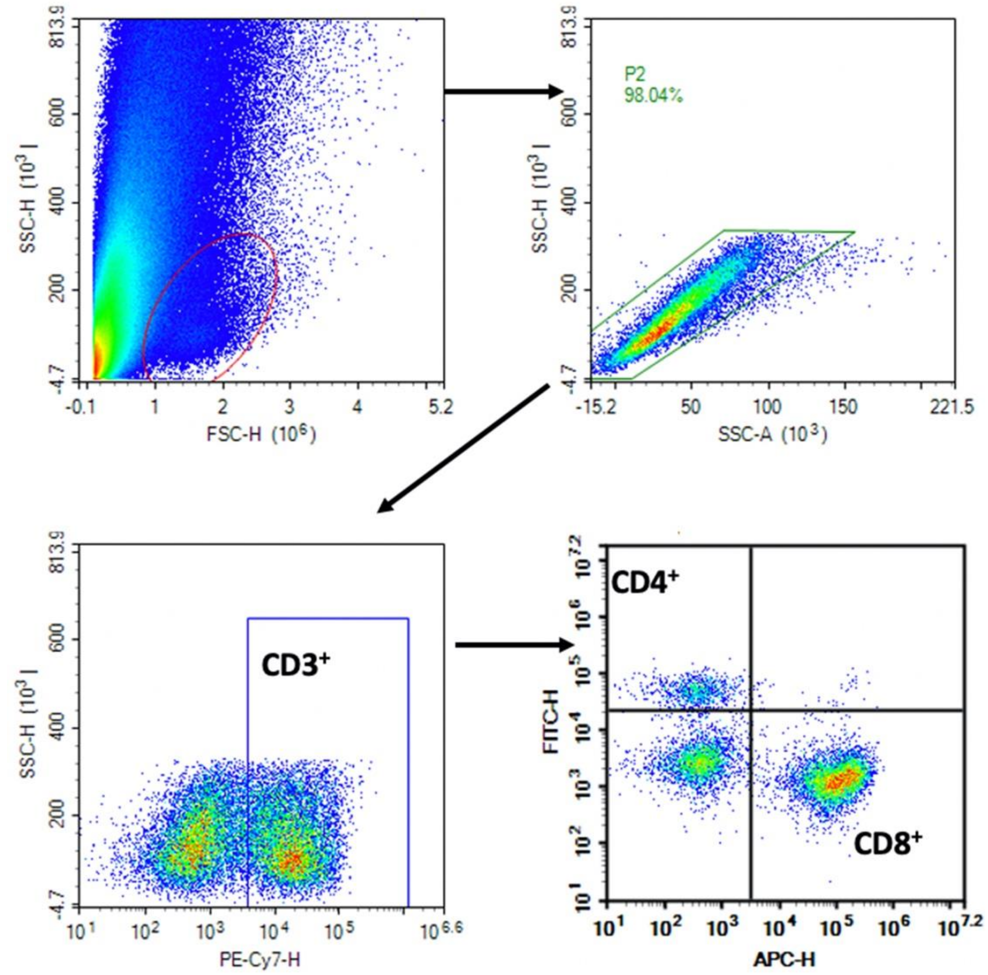

Figure S16. Representative scatter plots and gating information derived from analysis of CD3^{+} CD4^{+} and CD3^{+} CD8^{+} T cells in the tumor.

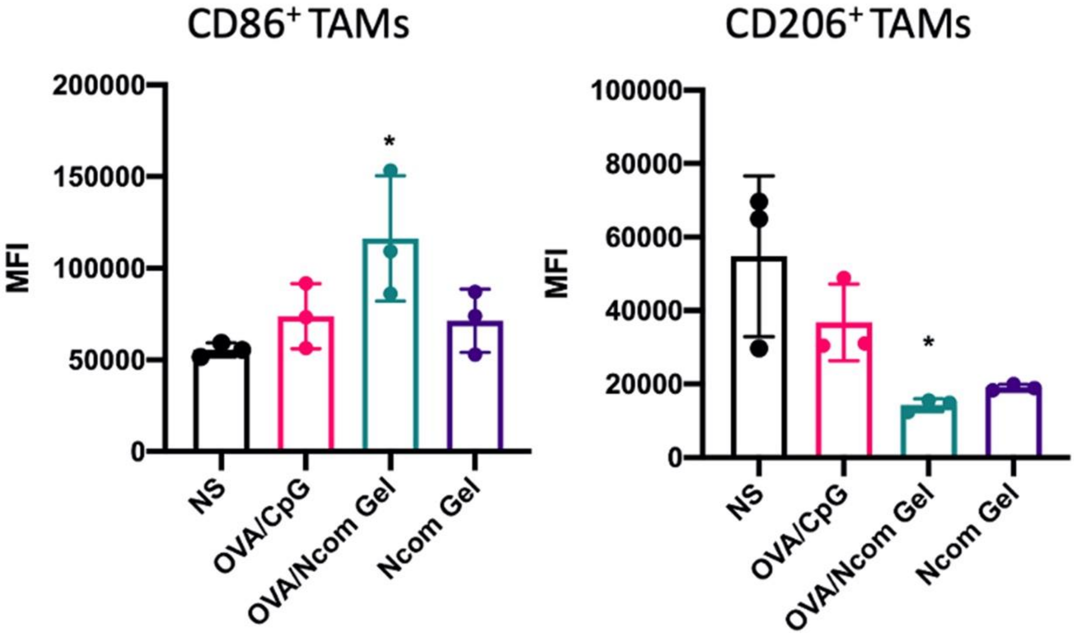

Figure S17. Fluorescence intensity of M1 TAMs (left) and M2 TAMs (right) were analyzed after various treatments (n=3 biologically independent samples, all data are represented as means ± s.d. and analyzed with one-way ANOVA with Tukey test. * P < 0.05, **P < 0.01, *** P< 0.001 and **** P< 0.0001).

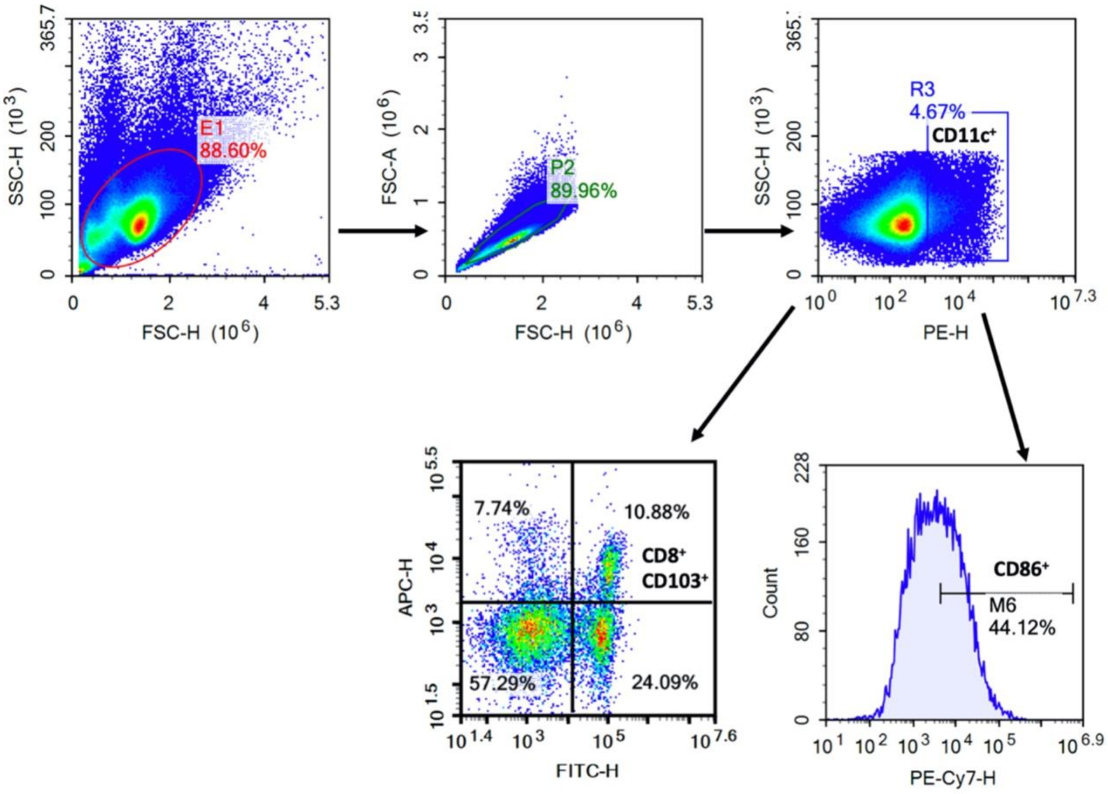

Figure S18. Representative scatter plots and gating information derived from analysis of CD11c$^+$ CD86$^+$ and CD11c$^+$ CD8$^+$ CD103$^+$ DCs in TDLN.

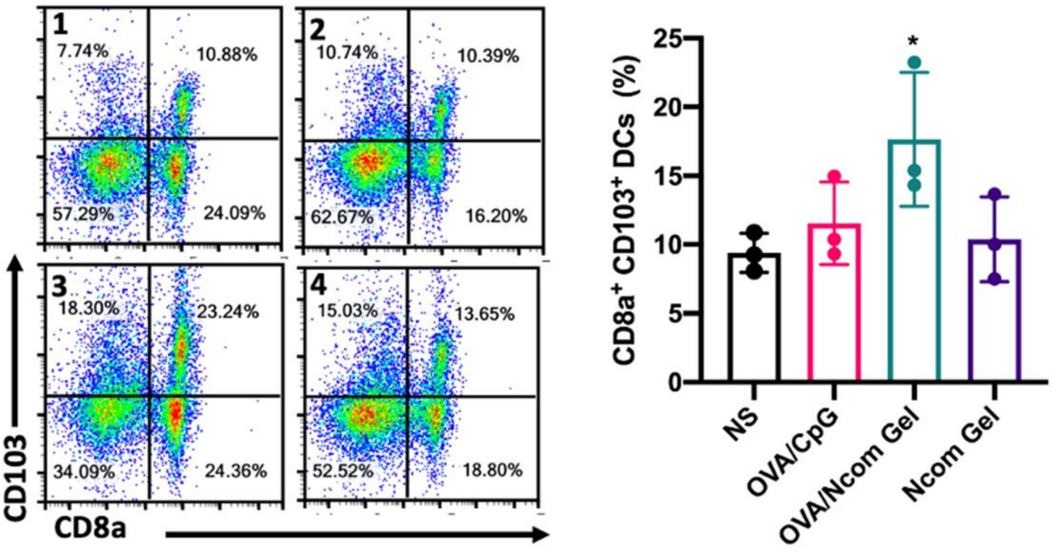

Figure S19. The representative flow cytometry dot plots of $CD8^+CD103^+$DCs in tumor-draining lymph node (TDLN) and quantitative data were shown (n=3 biologically independent samples, all data are represented as means ± s.d. and analyzed with one-way ANOVA with Tukey test. * P < 0.05, **P < 0.01, *** P< 0.001 and **** P< 0.0001).

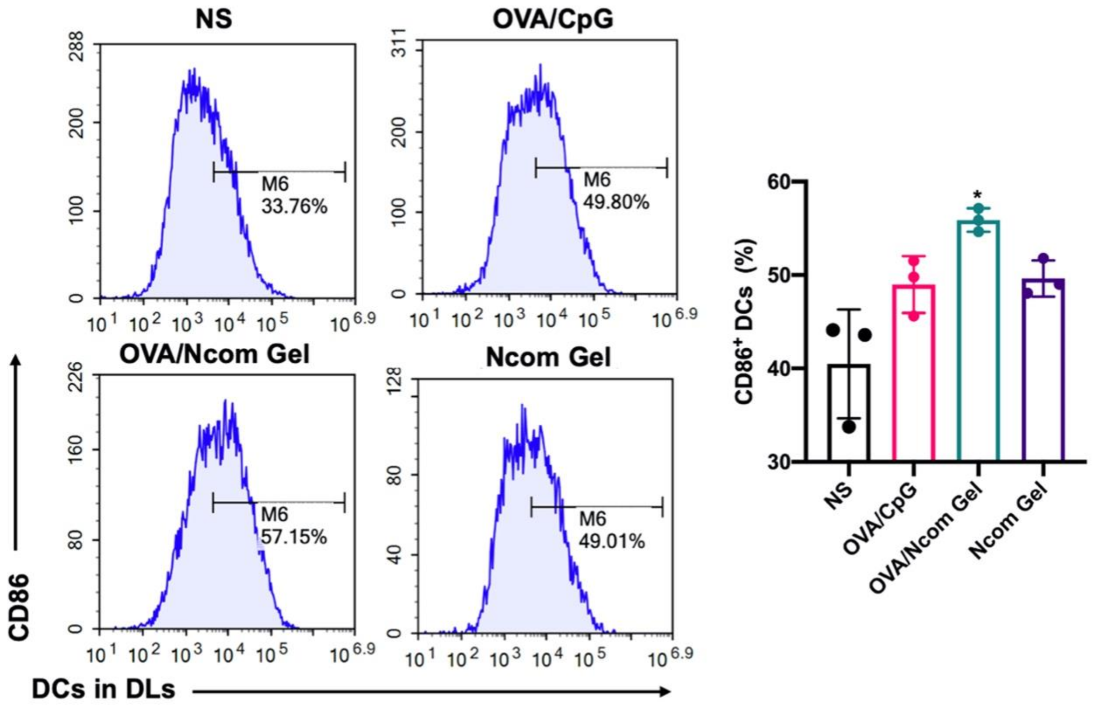

Figure S20. The representative flow cytometry histogram (left) and quantitative data (right) of CD86^{+}DCs in TDLN were analyzed after different treatments (n=3 biologically independent samples, all data are represented as means ± s.d. and analyzed with one-way ANOVA with Tukey test. * P < 0.05, **P < 0.01, *** P< 0.001 and **** P< 0.0001).

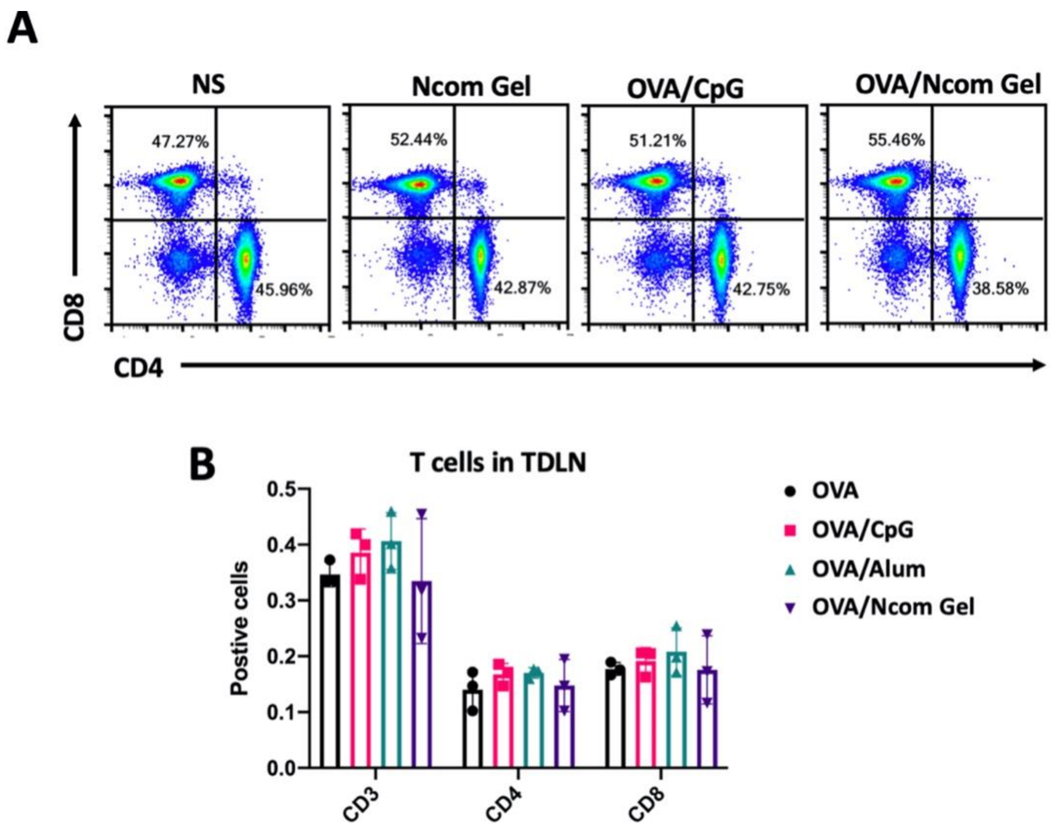

Figure S21. The representative flow cytometry dot plot of $CD8^+$ T cells and $CD4^+$ T cells in TDLN (top) and quantitative data (bottom) were examined after 2 days of treatment (n=3 biologically independent samples, all data are represented as means ± s.d.).
